# Wearable-Based Affect Recognition—A Review

**DOI:** 10.3390/s19194079

**Published:** 2019-09-20

**Authors:** Philip Schmidt, Attila Reiss, Robert Dürichen, Kristof Van Laerhoven

**Affiliations:** 1 Robert Bosch GmbH, Robert-Bosch-Campus 1, 71272 Renningen, Germany; 2 Ubiquitous Computing, Department of Electrical Engineering and Computer Science, University of Siegen, Hölderlinstr. 3, 57076 Siegen, Germany

**Keywords:** review, affective computing, affect recognition, wearables, data collection, physiological signals, machine learning, physiological features, sensors

## Abstract

Affect recognition is an interdisciplinary research field bringing together researchers from natural and social sciences. Affect recognition research aims to detect the affective state of a person based on observables, with the goal to, for example, provide reasoning for the person’s decision making or to support mental wellbeing (e.g., stress monitoring). Recently, beside of approaches based on audio, visual or text information, solutions relying on wearable sensors as observables, recording mainly physiological and inertial parameters, have received increasing attention. Wearable systems enable an ideal platform for long-term affect recognition applications due to their rich functionality and form factor, while providing valuable insights during everyday life through integrated sensors. However, existing literature surveys lack a comprehensive overview of state-of-the-art research in wearable-based affect recognition. Therefore, the aim of this paper is to provide a broad overview and in-depth understanding of the theoretical background, methods and best practices of wearable affect and stress recognition. Following a summary of different psychological models, we detail the influence of affective states on the human physiology and the sensors commonly employed to measure physiological changes. Then, we outline lab protocols eliciting affective states and provide guidelines for ground truth generation in field studies. We also describe the standard data processing chain and review common approaches related to the preprocessing, feature extraction and classification steps. By providing a comprehensive summary of the state-of-the-art and guidelines to various aspects, we would like to enable other researchers in the field to conduct and evaluate user studies and develop wearable systems.

## 1. Introduction

Affect recognition aspires to detect the affective state (e.g., emotion or stress) of a person based on observables. Hence, from a theoretical point of view, affect recognition can be seen as a signal and pattern recognition problem [1]. From a practical standpoint, affect recognition is an essential building block of affective computing, which aspires to develop devices, which are able to detect, process and interpret human affective states. As a result, affect recognition is a highly interdisciplinary research field with links to signal processing, machine learning, psychology and neuroscience.

The experiments of Bower [2] indicate that decision making and memorisation of a person are strongly influenced by their affective states. Therefore, a holistic user model requires the affective state as an integral part. Such a model could not only provide reasoning for the user’s actions but also be of great value to the user by providing insights into his/her affective states. Correlations between certain affective states (e.g., joy) and places (e.g., cinema) or persons (e.g., friends) could be helpful for users when planning their leisure activities. From a healthcare point of view, stress is a particularly interesting affective state. This is due to the severe side effects of long-term stress, which range from headaches and troubled sleeping to an increased risk of cardiovascular diseases [3,4,5]. According to the British Health and Safety Executive (HSE), stress accounted for 37% of all work-related ill health cases in 2015/16 [6]. As a result, a frequently pursued task in affect recognition is to build automated stress detection systems.

In the affect recognition literature, numerous approaches based on audio-visual data [7,8], contextual cues [9], text [10], body postures [11] and physiology [12,13,14] have been presented. In this review, we focus on approaches utilising wearable sensors (recording mainly physiological and inertial parameters). The reasons for this focus are twofold: First, due to their rich functionality and form factor, wearables like smartphones/watches are popular among users. A clear goal of affect recognition systems is to be applicable in everyday life. Such wearable-based affect recognition could, for instance, provide users with data driven insights into their affective spectrum by linking certain states (e.g., stress) to locations (e.g., office). Due to their computational power and integrated sensors, wearable devices are ideal platforms for many applications, e.g., counting steps, or estimating burned calories and recently a first generation of affect (e.g., stress) recognition systems entered in this sector [15]. Second, parameters observable with wearable sensors (such as changes related to the cardiac system or electrodermal activity) provide valuable insights related to the user’s affective state. Moreover, most related work relies on a multimodal setup. D’mello and Kory [1] pointed out that affect recognition systems basing their decisions on multimodal data tend to be almost 10% more accurate than their unimodal counterparts.

The aim of this work is to provide a broad overview and in-depth understanding of the theoretical background, methods and best practices in wearable-based affect and stress detection. By providing a comprehensive summary of the state-of-the-art, we would like to enable other researchers to conduct and evaluate user studies and develop novel wearable-based systems. Since the focus is on wearable solutions, approaches and studies relying mainly on audio, video, or text information are not subject of this review. Although affect recognition systems based on audio-visual data are very powerful and incorporated in products (e.g., Affectiva [16]), we exclude these modalities due to their limitations regarding mobile systems for everyday life and their intrusive nature. We refer readers with an interest in affect recognition or sentiment analysis methods based on audio or visual data to Poria et al. [17]. Moreover, work relying solely or mainly on smartphone data is excluded as well, since we focus on approaches relying on the observation of physiological changes of the user. Details concerning affect recognition based on smartphone usage can be found in Miller [18]. As outlined above stress detection is a pressing topic in many domains, for instance, Rastafoo et al. [19] recently reviewed the state-of-the-art in driver stress detection. Finally, we exclude the extensive amount of work done in the field of electroencephalogram-based (EEG) affect recognition due to the practical limitations of EEG in real-life scenarios. EEG-based affect recognition is reviewed, for instance, by Kim et al. [20].

The rest of this review is organised as follows. In Section 2, psychological models of affect are presented. Then, the influence of different affective states on the human physiology and the sensors commonly used to measure physiological states and changes are detailed in Section 3. Next, guidelines for laboratory and field studies are presented in Section 4. For this purpose, we outline standardized lab protocols eliciting affective states and address the issue of ground truth generation in the field. Furthermore, Section 4.3 details publicly available datasets, containing wearable-based sensor data. Section 5 outlines the standard data processing chain employed in affect recognition, focusing on preprocessing, feature extraction and classification. Finally, this work is concluded in Section 6 by summarising the main findings and outlining future challenges in wearable-based affect recognition.

## 2. Interdisciplinary Background

In this section an overview of the terminology used in affect recognition (AR) will be provided. For this purpose different psychological and physiological constructs of affective states will be presented and summarized.

### 2.1. Working Definitions of Affective Phenomena

In order to tackle AR working definitions of different affective states are required. Psychologists have been studying human emotions intensively. Hence, the emotional models and terms employed in AR are “borrowed” from psychology. In this section terms commonly used in AR are defined and models for emotions and stress are introduced.

Despite a growing body of research, it is still difficult to define the terms affect, emotion and mood in a precise way. Below working definitions are provided and differences between the constructs are highlighted. Russell [21] defines affect as a neurophysiological state. This neurophysiological state is consciously accessible as simple raw (nonreflective) primitive feeling [22]. Affect is not directed at a specific event or object and lasts only for a very short time. In contrast, emotions are intense and directed feelings, which have a short duration. Emotions are an indicator of affect and arise from a cognitive process evaluating a stimulus (e.g., a specific object, an affect, or a thought). Hence, emotions are directed at a stimulus. To illustrate these aspects, Liu [22] uses the example of watching a scary movie: If you are affected, the movie elicits the feeling of being scared. The mind processes this feeling (*scared*), adds an evaluation (*‘this is really spooky’*) and expresses it to you and your surroundings as an emotion (*fear*) by, for example, crying [22]. In the AR literature, the terms mood and emotion are often used interchangeably. However, in contrast to emotions (and affects), mood is commonly defined to be less intense, more diffuse and to last for a longer time period. This difference between mood and emotion is best illustrated by considering the following example—One can get angry very quickly but it is hard to stay angry for a longer time period. However, the emotion *anger* might lead to an *irritable* mood, which can last for a long time [22].

In the remainder of this review the term affective state will be used to describe the internal state of a person, which can be referred to as emotion, mood, and/or affect.

### 2.2. Emotion Models

In this section emotional models frequently employed in AR literature are detailed. These are grouped into two distinct types:

1. **Categorical models:** Here different emotions are represented in discrete categories.
2. **Dimensional models:** Following this approach, emotions are mapped into a multidimensional space, where each of the axis represents a continuous variable.

**Categorical models** date back to ancient Greek and Roman philosophers [17]. Cicero, for instance, distinguished four basic categories of emotions, namely *fear*, *pain*, *lust* and *pleasure* [23]. Darwin [24] also conducted studies on emotions and came to the conclusion that emotions have an evolutionary history and, hence, are shared across cultures. Similar to Darwin [24], Ekman [25] argues that basic emotions are shared across cultures and appear to be universally recognised. Following Ekman and Friesen [26], six basic emotions can be distinguished: *joy*, *sadness*, *anger*, *fear*, *disgust*, and *surprise* [26,27]. These basic emotions are discrete and have distinct physiological patterns, for example, facial muscle movement. Being able to express basic emotions can be attributed with a number of (evolutionary evolved) physiological and communicative functions: *Disgust*, for example, is often expressed by a certain facial expression and a wrinkled nose. On a physiological level this facial expression limits inhalation of malodorous particles. On the communicative level, this distinct facial expression, performed for instance as reaction to rotten food, has the potential to warn others.

In 1980, Plutchik [28] developed another taxonomy to classify discrete emotions. The so-called ’wheel of emotions’ comprises of eight primary emotions: *grief, amazement, terror, admiration, ecstasy, vigilance, rage*, and *loathing*. Following Plutchik [28], the primary emotions mix and give rise to more complex emotions. In addition, emotions are expressed at different intensity levels. In the domain of wearable AR, categorical models were for instance used by Zenonos et al. [29]. In their study the authors presented an approach to distinguish eight different emotions and moods (*excited*, *happy*, *calm*, *tired*, *bored*, *sad*, *stressed*, and *angry*).

**Figure 1:**
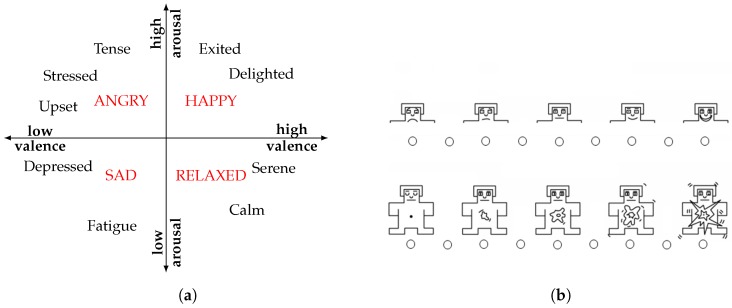
Exemplary dimensional model. (**a**) Schematic representation of the circumplex (valence-arousal) model. Adapted from Valenza et al. [36]; (**b**) Exemplary Self-Assessment Manikins [38], used to generate labels in the valence-arousal space. Adapted from Jirayucharoensak et al. [40].

The above presented model of basic emotions is not unquestioned and one point of criticism is that some languages do not have words for certain basic emotions [30]. According to Reference [31], in Polish, for instance, there is no exact translation for the English word *disgust*. **Dimensional models** where emotions are mapped into a multidimensional space, mitigate this shortcoming. The first dimensional approach dates back to Wundt [32], who describes momentary emotions as a single point in a three-dimensional space [33]. Wundt’s emotional space is spanned by the pleasure-displeasure, excitement-inhibition and tension-relaxation axes. At the end of the 1970s, Russell [30] postulated a two-dimensional model, namely the circumplex model (see Figure 1a). This model has been very impactful and in the circumplex model, affective states are represented as discrete points in a two-dimensional space, spanned by the axes valence and arousal. The valence axis indicates the perception on how positive or negative the current affective state is. On the arousal axis, the state is rated in terms of the activation level, for example, how energised or enervated one feels. The four quadrants of the circumplex model (low arousal/low valence (LALV), low arousal/high valence (LAHV), high arousal/low valence (HALV) and high arousal/high valence (HAHV)) can be attributed with *sad*, *relaxed*, *angry*, and *happy*. By adding further orthogonal axes, for example, dominance, the circumplex model is easily extended. In AR, the circumplex model and its variants are frequently employed [34,35,36,37]. Using the Self-Assessment Manikins (SAM) [38], the circumplex model can easily be assessed. These Manikins offer an easy graphical way for subjects to report their current affective state (see Figure 1b). In addition, the SAM are easily understood across cultures, due to their simple graphical representation. Another possible reason for the popularity of dimensional models in AR might arise from a machine learning (ML) point of view. The (at least two) independent axes of the circumplex model offer an interesting set of different classification tasks: The valence and arousal axes, for instance, can be binned into multiclass classification problems, for example, low/medium/high arousal or valence. In addition, posing classification problems based on the four quadrants named above is a frequently pursued task in AR, see for instance References [34,39].

### 2.3. Stress Models

In everyday life, *stress* or *being stressed* are terms used to describe the feeling of being under pressure. Stress is commonly elicited by an external and/or internal stimulus called stressor. However, from a scientific point of view, stress is primarily a physiological response. At the beginning of the 20th century, Cannon [41] coined the terms homeostasis and “fight or flight” response. Homeostasis describes a balanced state of the organism where its physiological parameters stay within an acceptable range (e.g., a body temperature of 37°C). Following Reference [41], both physiological and psychological stimuli can pose threats to homeostasis. Stressors can be seen as threats, disrupting homeostasis. In order to maintain homeostasis, even under extreme conditions, feedback loops (e.g., a fight or flight response) are triggered [41].

In the 1970s, Selye [42] defined stress to be/result in a ‘nonspecific response of the body to any demand upon it’. Following this definition, ‘nonspecific’ refers to a shared set of responses triggered regardless of the nature of the stressor, for example, physical or psychological. Recent stress models, for instance McEwen and Stellar [3], incorporate multiple effectors and advocate that the stress response is to some degree specific. The stress response is mainly influenced by two aspects: first, the stressor itself and, second, the organism’s perceived ability to cope with the posed threat [43]. Depending on the coping ability of the organism and estimated chances for success, eustress (positive outcome) and distress (negative outcome) are distinguished [44]. Eustress can have a positive (motivating) effect, while distress is perceived to be hindering (feeling worried or anxious). In order to illustrate this the following example can be used: Assume a person has to take an exam. Here, this exam represents an external stressor and the body reacts with a physiological stress response, for example, by increasing the blood glucose level. If the person feels well prepared for the exam and is looking forward to the challenge ahead, this can be interpreted as eustress. In contrast, if the person is not well prepared and feels like failing the exam, this can result in distress. Considering wearable stress recognition, distinguishing between eustress and distress is a largely unsolved problem due to the lack of adequate physiological indicators. However, long-term stress in general is associated with many severe health implications ranging from troubled sleeping and headaches to an increased risk for cardiovascular diseases [3,4,5]. Due to these severe side effects of long-term stress, the detection of stress is a frequent task in AR—Mozos et al. [45], Plarre et al. [46], Schmidt et al. [47], for instance target binary stress recognition tasks (*stress* versus *no stress*) and Gjoreski et al. [13] aimed at distinguishing different levels of stress (*no stress* versus *low stress* versus *high stress*).

Above different emotion and stress models were summarised. Although stress is not an emotion, a link between dimensional models and stress is readily established: Following Sanches et al. [48], a direct link between stress and arousal can be drawn. Valenza et al. [36] maps stress into the high arousal/negative valence (quadrant II) of the circumplex model (see Figure 1a). Following Thayer [49] and later Schimmack and Reisenzein [50], the arousal dimension of the ‘classical circumplex’ model can be split into tense arousal (stressed-relaxed) and energetic arousal (sleepy-active). According to Schimmack and Reisenzein [50], this split is justified by the observation that only the energetic arousal component is influenced by the sleep-wake cycle. Considering the wearable affect and stress recognition literature, a recent study conducted by Mehrotra et al. [51] uses this three-dimensional emotion model (valence, tense arousal and energetic arousal) to investigate correlation and causation between emotional states and cell phone interaction.

## 3. Physiological Changes and Objective Measures

In this section the affect-related changes in physiology and devices to measure these are presented. Section 3.1 provides background on the physiological changes and in section Section 3.2 commonly used sensors are presented.

### 3.1. Affective States and Their Physiological Indicators

Affective states and physiological changes are clearly linked, for example, if someone cracks a good joke we laugh or at least smile. With this physiological response we express *amusement*. Negative emotional states have even stronger physiological indicators. For instance, when being *afraid* or *anxious* one might start sweating, get a dry mouth, or feel sick.

Stress was characterised primarily as a physiological response to a stimulus, see Section 2.3. The most severe physiological reaction to a stressor is the so called ‘fight or flight’ response [41]. During this response the body prepares for a severe action, like fight or flight, releasing a mixture of hormones, like cortisol and adrenaline. This leads, for instance, to an increased breathing/heart rate, pupil dilation and muscle tension. The induced physiological responses are quite distinct and are a good example for the link between affective states and physiological changes.

Above the link between affective states and physiological responses was established using examples. The direction/causality, for example, do affective states cause physiological changes or vice versa, is still an open research question: At the end of the 19th century [52] postulated, that physiological changes precede emotions and that emotions arise from these changes. This is best illustrated considering the following example: Picture someone encountering a gigantic poisonous spider. After this encounter, the heart rate and the activity of the sweat glands of the subject would increase. Following the James-Lange-Theory, these physiological changes are not symptoms of *fear/disgust* but rather involuntary physiological responses. According to James [52] these physiological responses, become an emotion/feeling, like *fear/disgust*, once a cognitive evaluation occurred. Hence, the subject could describe the process as “I feel afraid, because I have a racing heart”. This theory is supported, for instance, by experiments conducted by Levenson et al. [53], who found evidence that performing voluntary facial muscle movements exhibit similar changes in peripheral physiology as if the corresponding emotion is experienced. For instance, when the subjects were asked to make an angry face the heart rate was found to increase. This theory, of course, is not unchallenged. Following common sense, a stimulus is perceived, it elicits an feeling and the physiological responses are triggered. Hence, the subject could describe the process as “I have a racing heart, because I’m afraid of the poisonous spider”. Following the Cannon-Bard-Theory, the perceived stimulus is processed in the brain and then the physiological response and affective states arise simultaneously [54]. Hence, the subject could describe the process as “The spider makes me feel afraid and I have a racing heart”. The debate outlined above is, from a theoretical point of view, very interesting. However, it is out of scope of this review. Wearable-based AR, utilizes these affect-related changes in physiology.

SNS PNS Affective states occur spontaneously and are accompanied by certain physiological pattern. These physiological responses are hard or even impossible to control for humans. The **autonomic nervous system (ANS)** directs these unconscious actions of the organism. Hence, the ANS plays a key role in directing the physiological response to an external (e.g., event) or internal (e.g., thought) affective stimulus. The ANS has two major branches: the sympathetic nervous system (SNS) and the parasympathetic nervous system (PNS). In Table 1, the key contributions of the SNS and PNS are displayed. As the SNS is mainly associated with the ‘fight or flight’ response, an increased activity of the SNS indicates high arousal states. In other words, the main function of the SNS is to provide energy by increasing a number of physiological parameters (e.g., respiration rate, glucose level, etc.). The PNS, in contrast, regulates the ‘rest and digest’ functions [55].

The interplay of sympathetic nervous system (SNS) and parasympathetic nervous system (PNS) is best illustrated considering the cardiovascular system. In reaction to a potential threat, the SNS increases the heart rate (HR). Once the threat is over, the PNS reduces the HR, bringing it back to normal [56]. A common measure to quantify the interaction of SNS and PNS is the **heart rate variability (HRV)**. The HRV is defined as the variation in the beat-to-beat intervals. An increased/decreased HRV indicates increased activity of the PNS/SNS, respectively. As a result, the HRV is a rather simple but efficient measure to quantify the contributions of the PNS/SNS. Hence in related work, the HRV is employed to detect stress [56]. Changes in the **electrodermal activity (EDA)** are another simple but effective measure to assess the SNS activity, too. This is due to the fact, that changes in EDA are governed by the SNS [56]. Hence, following Dawson et al. [57] the EDA is particularly sensitive to high arousal states, like *fear*, *anger*, and *stress*. EDA has two main components, namely the skin conductance level (SCL) and the skin conductance response (SCR). The SCL, also known as tonic component, represents a slowly varying baseline conductivity. In contrast, the SCR, also called phasic component, refers to peaks in the EDA signal. For most other vital parameters, the contributions of PNS and SNS are more interleaved. Hence, their responses are less specific. Nevertheless, also considering **respiration and muscle activity**, certain patterns can be attributed to different affective states. For instance, the respiration rate increases and becomes more irregular when a subject is more aroused [34]. Later, in Section 5.2, a detailed description of physiological features will be provided.

As outlined above, the SNS contributions to high arousal states are quite distinct. In a recent meta analysis, Kreibig [58] investigated the **specificity of the ANS** response to certain affective states. A subset of these findings, including two positive and two negative affective states, is presented in Table 2. Considering for instance *anger*: a majority of the analysed studies showed that it coincides with an increased heart rate (HR), skin conductance level (SCL), number of skin conductance response (SCR)s and a higher breathing rate. Since *anger* represents a high arousal state, governed by the SNS, these reactions were expected. Non-crying *sadness* was found to decrease HR, SCL and number of SCRs, while increasing the respiration rate. In the circumplex model (see Figure 1a), *sadness* is mapped into the third quadrant (low valence, low arousal). Hence, the arousal level is expected to drop which is confirmed by Table 2. *Amusement* and *happiness* are both positive affective states with a similar arousal level. Hence, it is not surprising that they have a similar physiological fingerprint. For more details, we refer the reader to Kreibig [58].

The findings of Kreibig [58] suggest that affective states have certain physiological fingerprints which are to some degree specific. These findings are promising, as they indicate that distinguishing affective states based on physiological indicators is feasible. However, in the context of **wearable-based AR**, the following aspects should be considered [59]:

1. Physiological measures are *indirect* measures of an affective state.
2. Emotions are subjective but physiological data are not.
3. Although some physiological patterns are shared across subjects, individual responses to a stimulus can differ strongly.
4. Multimodal affect detecting systems reach higher accuracies than unimodal systems [1].
5. The physiological signal quality often suffers from noise, induced by motion artefacts and misplacement.

### 3.2. Frequently Employed Sensors

This section provides an overview of the sensor modalities frequently employed in wearable-based AR. The clear aim of AR is to find robust methods assessing the affective state of a user in everyday life. Hence, a major goal is to use sensor setups which are minimally intrusive and pose only minor limitations to the mobility of the user. As detailed in Table 1 and Table 2, physiological changes in the cardiac system and electrodermal activity are key indicators for affective states. Therefore, most studies utilise these modalities. Nevertheless, sensors measuring other physiological parameter, like respiration or muscle activity, can also contain valuable information on the affective state of a person [58]. Table 3 lists the most relevant sensors, grouped according to their placement on the human body. Below, each of the listed modalities is discussed, detailing advantages and limitations.

#### 3.2.1. Cardiac Activity

In order to assess the heart rate (HR), heart rate variability (HRV) and other parameters related to the cardiac cycle, the electrocardiogram (ECG) serves as gold standard. For a standard three-point ECG, three electrodes are placed on the subject’s torso, measuring the depolarisation and repolarisation of the heart tissue during each heartbeat. ECG samples are collected with frequencies up to 1024 Hz. However, when acquired with such high frequency the signal can be downsampled to 256 Hz without loss of information [31]. Furthermore, experiments of Mahdiani et al. [60] indicate that a 50 Hz ECG sampling rate is sufficient to obtain HRV-related parameters with a reasonable error. Using photoplethysmogram (PPG) also provides information about the cardiac cycles. In contrast to ECG, PPG utilises an optical method: The skin voxel, beneath the sensor, is illuminated by a LED and a photodiode measures the amount of backscattered light. Alternatively if the detector is on the opposite side of the respective body part (e.g., fingertip or earlobe), the amount of transmitted light is measured. Hence, the cardiac cycle is captured by the PPG signal, where the pulsatile part of the PPG signal reflects the pulsatile component in arterial blood flow [61]. Data obtained from a PPG sensor tends to be noisier than ECG data. This is due to artefacts caused by motion, light from external sources, or different skin tones, which influence the reflection/absorption properties of the skin. PPG sensors can be attached to the ear, wrist [13] or the finger tip [62] of subjects. The PPG modality finds broad application in fitness trackers and smartwatches, which can be attributed to the small form factor of the sensory setup. Typical sampling rates of PPG devices are below 100 Hz.

EDA

#### 3.2.2. Electrodermal Activity

The electrodermal activity (EDA) is commonly measured at locations with a high density of sweat glands, for example, palm/finger [56] or feet [63]. Alternative locations to measure an EDA signal are the wrist [13] or the torso [64]. In order to assess EDA, the resistance between two electrodes is measured. From a technical point of view, EDA data is recorded employing either constant-current (measuring skin *resistance*) or constant-voltage systems (recording skin *conductance*) [57]. However, due to the more linear relationship between the skin conductance and the number of active sweat glands, Lykken and Venables [65] argues strongly for a direct measure of the skin conductance using constant-voltage systems [57]. In recent AR research the *Empatica E4* is a frequently employed device to collect EDA data [13,64,66,67]. Having the form factor of a smartwatch, the E4 samples the EDA signal at 4 Hz, which is sufficient to distinguish the SCR from the SCL. Although the EDA is strongly influenced by the SNS, external parameters such as humidity, temperature, or the physical activity have a strong influence.

#### 3.2.3. EMG

Muscle activity is measured using surface electromyogram (EMG). For this purpose, a pair (or array) of electrodes is attached to the skin above the muscle under consideration. The electrical potential is generated when the muscle cells are activated and the surface electrodes are used to recorded changes in the electric potential. The frequency range of the muscle activity ranges from 15 to 500 Hz [68]. Hence, in order to capture the full spectral range, the minimal sampling rate of the EMG modality should be around 1000 Hz. One source of noise in surface EMG are potential changes in adjacent muscles and cardiac activity. Depending on the measurement position, the QRS complex (indicating depolarization of the cardiac ventricles and the following contraction) can cause artefacts which require postprocessing beyond normal filtering. Considering related work in AR literature, EMG electrodes are often placed in the face (e.g., on the zygomaticus major [35]) or on the shoulder (e.g., on the upper trapezius muscle [34,35,69]).

RIP

#### 3.2.4. Respiration

Although respiration can be assessed indirectly from measuring the blood oxygen level, a direct measurement contains more information about the actual respiration pattern. Commonly, a chest belt (respiratory inductive plethysmograph (RIP) [46]), which is either worn thoracically or abdominally, is utilised to measure the respiration pattern directly. During a respiration cycle (inhalation and exhalation), the thorax expands and constricts. Hence, the chest belt experiences a sinusoidal stretching and destretching process, from which different physiological parameters like respiration rate and volume can be derived. Healey and Picard [63] sampled their respiration sensor at 31 Hz. However, following the Nyquist theorem a lower bound on the sampling rate of a RIP setup can be around 10–15 Hz. Nowadays, chest belts are mainly used by athletes monitoring their training progress. However, these devices have not found broad applications outside this domain.

#### 3.2.5. Skin-Temperature

As the blood flow to the extremities is restricted during a ‘fight or flight’ response, changes in peripheral temperature is an interesting parameter. These changes in skin-temperature (TEMP) can be measured using either an infrared thermopile or a temperature-dependent resistor. A common confounding variable for body temperature measurements is the ambient temperature, which can have a strong influence on the recording depending on the location of the thermopile. As changes of the body temperature are low-frequent, a sampling rate of 1 Hz is sufficient.

#### 3.2.6. EEG and EOG

EEG EOG PD The physiological modalities detailed above are only minimally intrusive. Hence, they are frequently employed in AR lab and field studies [56,63,70,71]. In addition to the modalities listed above electroencephalogram (EEG) and electrooculography (EOG) are also often applied in AR studies. EEG, measuring the ionic current of brain neurons using electrodes placed on the scalp, was for instance employed by Soleymani et al. [72] to detect video-elicited emotions. EOG, which records horizontal and vertical eye movements by placing electrodes above/below and left/right of the eye, has been used by Koelstra et al. [35]. In our opinion, these modalities have the following disadvantages:

- Both require the placement of electrodes on face/scalp. Hence, EEG and EOG are quite intrusive and not practical for everyday life.
- They pose strong limitations on the movement of the participants and, hence, are not really applicable in real world scenarios.
- EOG and EEG are prone to noise generated by muscle activity.

Therefore, in the remainder of this review EEG and EOG will be given very little attention.

#### 3.2.7. Inertial Sensors

Inertial sensors, incorporating a 3-axes acceleration (ACC), gyroscope and magnetometer, are commonly used in human activity recognition (HAR). In AR field studies the ACC signal can provide context information about the physical activity of the user. Gjoreski et al. [13], for instance, used ACC data to classify six different activity types (*lying*, *sitting*, *standing*, *walking*, *running* and *cycling*). These activities, were then used as an additional input into a stress detection system. This certainly highlights the value of contextual information. However, results of Ramos et al. [73] indicate that in order to detect stress it is sufficient to estimate the intensity level of an activity instead of performing an exact activity classification.

#### 3.2.8. Context

Finally, following Muaremi et al. [74], smartphones offer an ideal platform to collect context information. This contextual data is aggregated by utilising position (GPS), sound snippets, calendar events, ambient light and user interaction with the phone [45,74,75].

Table 4 summarises recent wearable-based AR studies aspiring to detect different affective states, using wearable-based data. In order to identify relevant studied, a keyword-based search was performed in archival databases for the keywords affective computing/recognition, stress/emotion detection physiology and wearable-based AR, while explicitly removing non-wearable and EEG-only work. A detailed comparison of the employed classification algorithms, number of target classes, setting (e.g., lab or field), number of subjects, validation procedure and obtained accuracies, will be presented in Table 9. In the studies presented in Table 4, the target affective states are rather diverse: Almost 39% of the presented studies aimed to detect stress. For this purpose, different types of stressors (e.g., mental, physical, or social [46,76]) or different stress levels [13] are distinguished. Both the severe health implications and the strong physiological stress response (see Section 2.3), explain the popularity of stress recognition. According to Table 4, various studies aim to recognise different emotional categories, distinguishing up to eight different affective states. Dimensional models of emotions (e.g., valence-arousal space) were used in 37% of the analysed studies. In 15% of the considered studies EEG was recorded. Nevertheless, there exists a large body of work, utilizing EEG data to classify different affective states. However, as mentioned in Section 1 this modality is not in scope of this review. As a result, studies utilizing EEG data are given less attention here. Concluding from Table 4, sensor modalities monitoring cardiac activity are employed in 87% of the studies. EDA data was recorded in 76% of the studies. The popularity of these signals, certainly is linked to the strong impact of arousal-related changes on cardiac and electrodermal activity (see Section 3.1). In 32% of the considered studies, respiration data was acquired. Kim and André [34] pointed out that increased arousal can lead to an irregular respiration pattern. Finally, ACC, EMG and TEMP data were recorded in 32% of the studies. In summary, it is observed that sensors measuring parameters directly influenced by the SNS are most popular. Sensory setups recording less distinct changes are employed less frequently.

## 4. Affect-Related User Studies

Picard et al. [12] pointed out that, in order to generate high quality physiological data for affect detection, carefully designed study protocols are required. In order to reduce subject bias it might be necessary to disguise the true purpose of the study. However, if a deception is necessary for the protocol it is essential to uncover the true aim at the end of the protocol. Moreover, every study should be reviewed and approved by an ethics (or a similar) committee.

The arguably most important decision is whether the experiment is to be conducted in a laboratory setting or in the wild. A key issue when designing a field study is accurate label generation. In contrast, during a lab study, obtaining high quality labels is a minor issue as either the study protocol can be used or dedicated time slots for questionnaires can be reserved. However, considering lab studies, the desired affective states have to be elicited by a carefully chosen set of stimuli. If these stimuli are not appropriate, the desired effects might not occur. On the other hand, during field studies, affective stimuli do not have to be designed, as different affective states occur naturally. Section 4.1 provides an overview of protocols employed for user studies in the lab. Section 4.2 summarises related work on how to plan and conduct affect-related field studies, focusing especially on the employed questionnaires. Finally, as conducting an own user study is always a time consuming task, publicly available datasets are described.

### 4.1. Affect-Related User Studies in Laboratory Settings

Humans differ in their personality. Hence, generating data that corresponds to a particular emotional state is a challenging task [90]. However, due to the controlled lab environment, researchers can conduct studies following well-designed protocols. Another advantage of lab studies is that their replication is possible, due to the well defined experimental protocol. Below a detailed overview of stimuli frequently employed to elicit affective states in AR lab studies is provided:

**Images:** The International Affective Picture System (IAPS) [106] is a dataset comprised of colour photographs. The IAPS was compiled such that each image elicits an emotional reaction. Each image was rated multiple times by study participants, providing labels in the valence and arousal space. Mikels et al. [107] identified a subset of IAPS images, which elicits certain discrete emotions. Hence, depending on the desired emotion, one can choose particularly strong images from this subset. In the AR domain, the IAPS has, for instance, been used by Leon et al. [80] and by Hamdi et al. [90]. In the experiments presented by Leon et al. [80], 21 images from the IAPS were used to elicit three different affective states (*neutral*, *positive*, *negative*). Hamdi et al. [90] exposed their study participants to ten images from the IAPS and aimed at recognising six basic emotions (*disgust*, *joy*, *surprise*, *sadness*, *fear*, *anger*) based on physiological data.

**Videos:** According to Gross and Levenson [108], short audiovisual clips are very suitable to elicit discrete emotions. Hence, video clips are frequently employed as stimuli [31,35,37]. A common procedure to select a set of videos evoking certain target emotions is to chose them from a large pool of videos. The process of identifying the most appropriate subset often happens in two steps: First, the clips are watched and rated by a large number of individuals. Second, the clips which elicit a certain emotion most reliably are chosen as stimuli in the study [35,72]. Recently, Samson et al. [109] published a study on 199 short amateur clips which were rated by 411 subjects with respect to three affective categories (*neutral*, *positive*, *negative*). In AR literature, there are many examples where audiovisual clips have been used to elicit different affective states. Koelstra et al. [35] chose in their experiments music clips with a length of 60 s. After each stimulus, the progress was displayed and a 5 s baseline was recorded. Soleymani et al. [72] showed their participants 60 to 120 s long excerpts from movies and after each clip a short neutral clip (15 s) was displayed.

**Acted emotions:** In the above detailed protocols, emotions are event-elicited. Another way of generating affective states is to ask the subjects to purposefully elicit emotions, for example, act an emotion. For instance, Hanai and Ghassemi [110] asked the study participants to tell at least one happy and one sad story. Other researchers asked trained actors to perform certain emotions [111,112]. These types of approaches are frequently employed in sentiment analysis and emotion recognition from audio/video data.

**Game elicited emotions**: Another way to elicit a target affective state is to ask the subjects to perform a certain task. Using a Breakout engine and introducing a latency between the user’s input and the reaction in the game, Taylor et al. [113] elicited frustration in their study participants. Martinez et al. [93] used four different versions of a Maze-Ball game to generate pairwise preference scores. The scores were generated by asking the subjects which of two games felt more *anxious, exciting, frustrating, fun*, and *relaxing*.

**Affective states elicited by immersive media:** Advances in Virtual Reality (VR), like head mounted displays or VR-headsets open new possibilities to elicit affective states. Up to now these methods have not found broad application in wearable-based AR. However, this is a particularly interesting elicitation method as it allows to simulate close to real world scenarios, hence, offering optimal control, while retaining ecological validity. Such a method was for instance applied by Riva et al. [114], who used a head mounted display, showing three different VR environments (virtual parks), as an affective stimuli, eliciting a neutral, anxious and a relaxed affective state. More recently, Marín-Morales et al. [103] employed four architectural environments, displayed to the subjects via a VR-headset, to elicit different affective states, too.

**Stress inducing study protocols:** There are numerous protocols aiming at eliciting stress in the study participants. Mason [115] showed that in order to trigger a (physiological) stress response, the situation has to be either novel and/or unpredictable and/or beyond control for the subject [116]. Stressors frequently employed in the AR literature can be categorised as follows:

**C1**  *Social-evaluative Stressors:* A task creating a socially relevant situation for the subject. For example, performing a task in front of a panel which evaluates the subject.
**C2**  *Cognitive Stressors:* A task demanding significant mental engagement and attention. For example, performing an (challenging) arithmetic task under time pressure.
**C3**  *Physical Stressors:* A task creating a physically uncomfortable situation. For example, being exposed to extreme hot or cold.

A well-studied and frequently employed stress elicitation protocol is the *Trier Social Stress Test* [117]. The Trier Social Stress Test (TSST) has two conditions: a public speaking/job interview type of situation and a mental arithmetic task. Hence, the TSST incorporates both a social-evaluative (C1) and cognitive stressor (C2). Due to its reliability and easy set-up, the TSST was administered in numerous AR studies, e.g., Mozos et al. [45], Plarre et al. [46], Schmidt et al. [47], Hovsepian et al. [95], Gjoreski et al. [118]. Another stressor employed to target cognitive load is the so called *Stroop color test* [119]. In this condition, the subjects have to read out loud a sequence of colours written on a screen. However, the font colour does not match the written colour (e.g., green, blue, etc.). As a result, the task inflicts a high cognitive load and, hence, is a C2 stressor. The Stroop colour test has for instance been employed by Choi et al. [56], who aimed for the development of a wearable-based stress monitoring system.

Using *computer tasks*, stress can also be elicited reliable. Wijsman et al. [120], for instance, asked the subjects to perform a calculation, to solve a logical puzzle and to do a memorisation task. These tasks can all be seen as C2 stressors. These tasks had to be completed under time pressure. In addition, the subjects were distracted with sounds and parts of the protocol (memorisation task) were also recorded on video. Furthermore, as the participants of Reference [120] were told that their scores would be made available to their colleagues, the study protocol also had a social-evaluative component (see C1).

The *cold pressor* test, applied by Plarre et al. [46], can be used to evoke physical stress, corresponding to a C3 stressor. Following this test, the subjects are asked to place their hand into a bucket of ice cold water and leave it there for a predefined time (e.g., 60 s).

Now as a common set of stimuli has been detailed, the issue of **obtaining ground truth in a lab setting** is discussed briefly. Following for instance Plarre et al. [46], employed conditions (e.g., stressors) can be used as ground truth. One way to ensure the validity of the employed stimulus is to utilize *exactly the same* set up as in a related study. In addition, questionnaires integrated into the protocol should be used to verify that the desired affective states were successfully evoked (see for instance [47]). Typically, these questionnaires are used directly after each affective stimulus or condition. Ramos et al. [73], for instance, collected subjective stress levels after each stressor. In addition, the Stait-Trait Anxiety Inventory also has been used to capture different stress levels [13]. In order to generate labels in valence-arousal space the SAM are employed frequently [35,47,72]. In addition, as the perception of a stimulus can be influenced by **personality traits**, collecting this information, can be useful too [39].

### 4.2. Affect-Related User Studies in The Field

To develop affect-aware systems designed for everyday usage, data collection in the wild is essential. However, as the affective states occur naturally, the generation of a reliable ground truth has to be ensured differently. In this setting one can distinguish between questionnaires used in ecological-momentary-assessments (EMAs) and questionnaires employed during the pre- and post study phase. In the latter case constructs which are said to be constant for a longer time period (e.g., personality traits) are being queried. To assess the momentary affective state of a user, **EMAs**, also known as the experience sampling method, are employed. EMAs are a short set of questionnaires which the study participants file occasionally, to report their current affective state. Using EMAs, an important trade-off has to be considered. On one hand the affective state of the subject should be probed frequently. On the other hand, the subject should not be overloaded with questionnaires. The scheduling of EMAs can be either done *interval-based* (e.g., at certain/random times during the day) or *event-triggered*. In a study of Zenonos et al. [29], for instance, the subjects were prompted every two hours during their working hours. The EMAs employed, inquired eight different moods, asking for each the question *How have you been feeling for the last two hours?*. Another approach is to *distribute* a defined number of EMAs *randomly* over a time period. Muaremi et al. [74], for instance, divided the day into four sections and during each section subjects had to complete a randomly scheduled self-report. If the focus of a study lies on certain affective states or events, *event-triggered* self-reports can be utilized. In a study conducted by Hernandez et al. [88] call centre employees rated personal stress level after each call. Another example of event-based scheduling can be found by Rubin et al. [121]: Here subjects were asked to file an EMA once they became aware of the symptoms of a panic attack. In order to gain a deeper understanding of EMAs filed by the subjects daily screenings can be conducted [64]. Following Healey et al. [87], these screenings can be used to correct/extend participants’ annotations.

Besides the frequency of EMAs, the length and complexity of each single questionnaire are also important factors defining the burden for the subjects. In order to avoid overloading study participants, EMAs should focus on the main goal of the study and their completion should require only little effort.

In Table 5 questionnaires used during the pre- and post study as well as questionnaires employed in EMAs are displayed. As mentioned earlier the pre- and post study questionnaires, are used to aggregate information about longer time periods or traits of the subjects. Subjects’ **personality traits** can have an influence on their affective perception and physiological response [39].

Therefore, completing a personality-related questionnaire can provide valuable insights. These Big Five Inventory (BFI) personality traits were, for instance, used by Sano et al. [122] as features for predicting subjects’ mood. In addition, Taylor et al. [100] used personality traits to perform a groupwise personalization. Moreover, Wang et al. [9] used questionnaires assessing the mental health of their participants. For this purpose, the depression level (e.g., Patient Health Questionnaire (PHQ-9)) and loneliness level (UCLA loneliness scale) were recorded. As shown by Sano and Picard [92], Sano et al. [122], information on subjects’ **sleep quality** can be useful in affect-related studies. The Pittsburgh Sleep Quality Index (PSQI), inquiring information about the past four weeks, can serve as a suitable questionnaire for sleep behaviour and quality assessment. In order to assess the overall stress level of the study participants the Perceived Stress Scale (PSS), measuring the perception and awareness of stress, can be employed. The PSS has been used in field studies (e.g., References [9,92]) and in ambulatory setting [95]. The severity of stress-related symptoms can be scored using the Stress Response Inventory (SRI), or a simplified version of it, as shown by Kim et al. [82].

As detailed in Table 4, wearable-based AR studies, typically rely on well-known psychological constructs. Hence, in order to generate labels using EMAs these constructs are employed, too. However, standard questionnaires are often quite long and as a result not really applicable in EMAs. In order to mitigate this issue, standard questionnaires can be shortened, for example, using only a subset of items with the highest factor loads on the targeted construct. Such an approach was, for instance, presented by Muaremi et al. [74] using a shortened version of the Positive and Negative Affect Schedule (PANAS) as EMA, which consisted of five positive affect items (relaxed, happy, concentrated, interested and active) and five negative affect items (tired, stressed, sleepy, angry and depressed). One particularly frequently employed construct is the valence-arousal space. In order to generate **valence and arousal labels**, Healey et al. [87], for instance, used a tool called Mood Map, while Schmidt et al. [64] used the SAM. Furthermore, Wang et al. [9] used the Photo Affect Meter (PAM), assessing a similar construct. The PAM is implemented as smartphone app and the user selects from a set of 16 images the one that corresponds best to his/her current affective state. Zenonos et al. [29] provides an example for a custom EMA tool used for overall mood assessment: participants were asked to rate eight different moods on a scale from 0-100. The stress level of subjects can be assessed using a Likert-scale [13,88]. Moreover, the severity of a certain event can be scored using its’ symptoms. Rubin et al. [121], for instance, aimed to quantify the severity of panic attacks. Hence, they created a questionnaire including 15 panic attack symptoms. In case a panic attack occurred, subjects were asked to rate the severity of each of the 15 symptoms, using a severity rating of 1 (none) to 5 (extreme).

Historically, personal notebooks or journals were used for EMAs. However, these tools have been predominantly replaced by smartphone apps, as they offer an ideal platform to facilitate self-reports: Subjects do not need to carry a study-specific device, EMAs are automatically scheduled and uploaded, and contextual information available on the smartphone can be logged together with the ground truth information. A key to both frequency and completeness of EMA is participant’s motivation and using an appropriate **reward system** was proven to be beneficial: Participants of the study conducted by Healey et al. [87] received a base reward and an incremental reward, depending on the number of annotations made per day. Another reward structure was introduced by Wang et al. [9]: They offered all subjects a base reward, and the participants who completed most EMAs had the chance to win additional prizes.

In Table 6 an overview of recent **wearable-based AR field studies** is provided and the employed EMAs as well as their scheduling is summarized. This table illustrates that commonly a combination of pre-/post-study questionnaires are used. The pre-/post-study questionnaires can be employed as additional features or to group the participants [82,100]. In contrast, the data gathered via EMAs is often used as a subjective ground truth [13,96].

#### 4.2.1. Guidelines for Ecological-Momentary-Assessment

Based on the overview given above, we now provide practical guidelines for designing and applying EMAs in field studies. A similar analysis can be found in Schmidt et al. [64].

1. **Sampling rate**: When defining the number of scheduled EMAs over the observation period, the trade-off should between sampling as frequently as possible while not overloading the subject needs to be leveraged. A good compromise is to schedule an EMA every two hours [29] or approximately five times over the day [118].
2. **General scheduling**: A good practice is to schedule EMAs randomly. This ensures that the subjects are unprepared. If the EMAs shall be distribute approximately evenly over the observation, the following approach could be used: Divide the observation period into *N* sections (where *N* is the total number of EMAs over the observation period), and randomly schedule one EMA within each section. This approach was applied for example by Muaremi et al. [74]. Considering user studies in the lab, EMAs are typically scheduled directly after each affective stimulus or condition [47].
3. **Manual trigger**: As EMAs are commonly scheduled randomly during field studies, these questionnaires are independent of the participants’ affective states. Therefore, it is good practice to allow subjects to file an EMA (in addition to the generally scheduled ones) whenever they feel a change in their affective state. For example, Gjoreski et al. [13] enabled their study participants to log stressful events whenever they occurred.
4. **Number of items**: In order to avoid overloading subjects, the time required to answer an EMA should be minimized. Therefore, EMAs should focused on the goal of the study and include a minimal number items. A good compromise is to include at most ten items per scheduled EMA, as discussed by Muaremi et al. [74]. Considering lab studies, the length of an EMA is usually less critical: Here EMAs can be used during the cool-down phase after an affective stimulus, which allows the completion of longer EMAs.
5. **Situation labels**: It is important to generate labels on the spot and not in hindsight. This is due to memorization effects (e.g., halo effect), where the occurrence of a certain emotion can influence the perception of other affective states experienced during the observation period. Considering a field study, however, it is good practice to review the labels together with the study participant, for example, on a daily basis [87,95].
6. **Length of labels**: For a (mentally) healthy subject, affective states are expected to be stable on short time scales. However, when labels are generated using EMAs, the question arises how long these labels are valid. Considering lab studies, the labels generate using a questionnaire usually refer to the preceding stimulus (e.g., TSST). Considering field studies, however, the validity of labels is not as trivial. Depending on the focus of the study, one has to decide on a label length. If the study addresses mood, longer label periods, for example, 2 h [29], can be taken into account. If the study targets shorter affective states (e.g., emotions or stress), shorter label periods are used. For example, in order to detect and classify stress, Gjoreski et al. [13] considered ten minutes before and after each provided label.
7. **Ensure engagement**: Considering field studies, subjects motivation is key and keeping the subjects motivated will ensure high-quality labels, regarding both frequency and completeness. One way to boost motivation is an appropriate (incremental) reward system [9,87]. Another, way to increase subjects motivation might be to make the EMA optical appealing, for example, including graphical measures like the SAM or PAM.

### 4.3. Publicly Available Datasets

Conducting a user study is both a time consuming and a challenging task. However, there is a number of publicly available datasets. Depending on the research idea these datasets make the overhead of recording an own dataset obsolete. Furthermore, they facilitate benchmarking and allow a direct comparison of different approaches. Up-to-date the wearable-based AR community has only a handful of publicly available datasets containing data *solely* gathered via wearables. Therefore, we extend the scope of this section to datasets with a broader relevance to wearable AR. Below we present datasets which meet one of the following criteria: (a) being publicly available; (b) including data recorded from study participants being subject either to emotional stimuli or a stressor; and (c) including at least a few sensor modalities which can be (theoretically) integrated into consumer-grade wearables, which are applicable in everyday life. The datasets included in our analysis are summarized in Table 7. Considering the population column in Table 7 it becomes apparent, that the data available originates mostly from a young cohort of subjects. Only the dataset recorded by Taamneh et al. [134], features two different age groups, namely an elderly (age > 60) and a young group (aged btw. 18 and 28). This is certainly a limitation that needs to be considered when working with these datasets. Below we describe the datasets in detail.

The **Eight-Emotion** dataset [12] includes the data of one (female) study participant who was subject to the same set of stimuli over a time span of 20 days. The stimuli, a set of personally-significant imagery, were chosen by the subject to elicit the affective states *neutral*, *anger*, *hate*, *grief*, *platonic love*, *romantic love*, *joy*, and *reverence*. The physiological signals (ECG, EDA, EMG, and RESP) were sampled at 20 Hz. Major limitations of this dataset are: (a) only one subject is included, and (b) due to the low sampling rate aliasing artefacts are likely to occur.

**DEAP** (Database for Emotion Analysis using Physiological signals), recorded by Koelstra et al. [35], features physiological data of 32 study participants. In DEAP, one minute excerpts of music videos were used as stimuli. In total 40 clips were selected from a larger pool according to valence, arousal, and dominance ratings gathered during a pre-study. The physiological signals were all sampled with 512 Hz and later downsampled to 256 Hz. DEAP includes subjects’ ratings of the videos (valence, arousal, dominance, and liking). However, due to the employed protocol and the sensor setup, the DEAP participants were very limited in terms of movement. Therefore, one can expect that models trained on the DEAP dataset will have a limited performance in real-life settings.

The **MAHNOB-HCI** dataset, includes physiological data from 27 study participants (16 female) [31]. The dataset includes face and body video data from six cameras, data from an eye gaze tracker, and audio. The physiological data (ECG, EDA, EEG, RESP, and TEMP) was sampled at 1024 Hz. Apart from EEG data, the physiological data was downsampled to 256 Hz. The MAHNOB-HCI dataset includes data from two experiments: First, study participants watched a set of 20 video clips, each associated with an emotional keyword (*disgust, amusement*, *joy*, *fear*, *sadness*, and *neutral*). The goal of the second experiment was implicit tagging: Subjects were exposed to 28 images and 14 videos, and reported on the agreement with the displayed tags. For the AR community, especially the first experiment is of interest.

**DECAF** (DECoding user physiological responses to AFfective multimedia content) [37] was recorded in a laboratory setting with 30 subjects (14 female). The data recording consisted of two sessions for each subject, presenting music videos and movie clips, respectively. In the first session (music videos) the same set of clips as in DEAP were employed. For the second session, 36 movie clips were used as stimuli. From this pool of videos always nine correspond to a quadrant in the valence-arousal space. These 36 movie clips were selected from a larger pool during a pre-study based on valence-arousal ratings from 42 participants. For a detailed description, we refer the reader to Abadi et al. [37]. DECAF contains image (near-infrared face videos), magnetoencephalogram (MEG), and peripheral sensory data (ECG, EOG, and EMG). A clear limitation of DECAF is that, due to the MEG recordings, subjects were very restricted in their movements. Therefore, in contrast to real-life data DECAF is almost free from motion artefacts.

In **ASCERTAIN** (multimodal databASe for impliCit pERsonaliTy and Affect recognitIoN using commercial physiological sensors) [39], the same 36 movie clips as in DECAF were employed as stimuli. ASCERTAIN provides data from 58 subjects (21 female), and includes physiological modalities (ECG, EDA, EEG) as well as data recorded from a facial feature tracker. In addition, self-reports including arousal, valence, engagement, liking, and familiarity obtained for each video are included. Moreover, the dataset contains the Big Five personality traits for each subject. Hence, based on the recorded data, not only models predicting emotions can be created but also personality traits can be assessed.

**USI_Laughs** has been recently published by Di Lascio et al. [66]. The dataset contains physiological data recorded from 34 participants (6 female) recorded via an *Empatica E4* smartwatch (ACC, EDA, PPG, TEMP). Similar to prior work, funny clips were used to induce laughter. Following Di Lascio et al. [66] the main aim of the dataset is to facilitate the detection of laughter episodes based on physiological data. Here, the laughter episodes are to be considered as surrogate to positive emotions.

The **Driver stress** dataset [63] includes physiological data (ECG, EDA, EMG, RESP) from 24 participants. The dataset was recorded during one *rest* condition and two driving tasks (*city* streets and on a *highway* near Boston, Massachusetts). Depending on traffic the two driving tasks had a duration between 50 and 90 min. Using questionnaires and a score derived from observable events, the three study conditions (*rest, highway, city*) were mapped onto the stress levels low, medium, and high. Therefore, the dataset facilitates the development of real-life stress monitoring approaches. However, one limitation of the dataset is that the data was acquired at low sampling rates (e.g., EMG 15.5 Hz).

**Distracted Driving**, recorded by Taamneh et al. [134], includes multimodal (physiological and eye tracking) data from 68 subjects driving in a simulator on a highway. All participants were subject to four different distractions: no, emotional, cognitive, and sensorimotor distraction. As the dataset includes among other modalities EDA, heart and respiration rate. This data can be used to study the influence of different distractions on these parameter.

**Non-EEG** [76] is a dataset containing physiological data (EDA, HR, TEMP, SpO2, and ACC) from 20 subjects (4 female). The dataset was recorded during three different stress conditions (physical, cognitive, and emotional) and a relaxation task. Physical stress was evoked by asking the subjects to jog on a treadmill at three miles per hour. In order to elicit cognitive stress, the subjects had to count backwards from 2485 doing steps of seven. Lastly, emotional stress was triggered by anticipating and watching a clip from a zombie apocalypse movie. This dataset is particularly interesting as it contains only wearable-based data. Although the data collection was conducted in a lab setting, the subjects were (compared to the other datasets) less motion constrained due to the minimally intrusive nature of the sensors. However, a major limitation of the Non-EEG dataset is the low sampling rate of the employed devices (1 Hz and 8 Hz). In addition, as no ECG or PPG data was recorded, the HRV information can not be retrieved, a parameter shown to be relevant for stress recognition by various previous work (e.g., Kreibig [58]).

**StudentLife** [9] contains data from 48 college students (10 female). All participants were monitored over one academic semester (10 weeks). Unlike the afore described datasets StudentLife was recorded in the field. Considering the progress of the semester, it is expected that the students were more stressed towards the end of the data collection. This can be attributed to the examination period. StudentLife contains data recorded from the students’ smartphones (e.g., ACC, microphone, light sensor, and GPS/Bluetooth data). Moreover, various information related to the students’ context (e.g., class attendance) and smartphone usage (e.g., conversation frequency and duration) were recorded. In addition, StudentLife includes a large number of self-reports targeting physical activity, sleep, perceived stress, mood, mental well-being, and so forth. Due to the popularity of smartphones, the dataset is certainly of interest by facilitating affect and stress recognition purely based on smartphone usage patterns.’ However, a drawback of the StudentLife is that it does not include any physiological data.

**WESAD** (dataset for WEarable Stress and Affect Detection) is, to the best of our knowledge, the only publicly available dataset which contains data of subjects experiencing both an emotional and a stress stimulus [47]. WESAD includes data from 15 subjects (3 female) recorded in a laboratory setting. Each subject experienced three conditions: *baseline* (neutral reading task), *amusement* (watching a set of funny video clips), and *stress* (being exposed to the TSST). WESAD features physiological and motion data, recorded from both a wrist- and a chest-worn device. The following sensor modalities are included: ECG, PPG, EDA, EMG, RESP, TEMP, and ACC. Moreover, the high sampling rate (700 Hz) of the chest-worn device should be emphasised. Overall, WESAD is a fitting dataset for benchmarking affect and stress recognition algorithms based on physiological data.

## 5. Data Processing and Classification

In wearable-based affect recognition similar methods as in human activity recognition are employed. Following the classical time series analysis pipeline, presented by Bulling et al. [135], the raw data is first synchronised, filtered, segmented, features are computed, and finally feature-based classifiers are employed. The remainder of this section is structured as follows: In Section 5.1 the preprocessing of the raw data and segmentation is described. Section 5.2 provides an overview of features commonly used in wearable-based affect recognition (AR). The last step in the standard data processing pipeline is the classification. During this step a mapping between the computed feature and labels (e.g., emotion classes) is learned. Section 5.3 details common classification methods, applied validation schemes, and the results achieved in related work.

### 5.1. Preprocessing and Segmentation

When multimodal systems are employed, synchronisation of the different raw data streams might be necessary as a first step. Clear events, for example, pressing an event marker button or double tap gestures, can facilitate the synchronisation process. Depending on the transmission protocol of the recorded data, wireless data loss might be an issue. Different methods for handling missing values have been reviewed by García-Laencina et al. [136]. Omitting cases with missing data, is arguably the simplest of these method. However, it comes at the cost of losing a lot of information. Imputation, estimation of missing data points is another more elaborate approach.

A common step in preprocessing is to apply denoising filters, in order to improve overall signal quality. The type of filtering strongly depends on the respective sensor modality. Therefore, below an overview of the different filtering and further preprocessing techniques, applied to the modalities in scope (see Section 3.2) are detailed.

1. **3-axes acceleration Preprocessing:** A detailed analysis of preprocessing applied to ACC data can be found in Figo et al. [137]. In AR, the ACC data is often considered as a surrogate for the performed activity [13,45].
2. **Electrocardiogram Preprocessing:** In the raw ECG signal the R-peaks need to be identified. For this purpose, the Pan and Tompkin’s algorithm can be applied [138]. Once the R-peaks have been detected, the next step is to determine the RR intervals and assess their validity. For example, Hovsepian et al. [95] present an algorithm to assess the validity of candidate RR intervals. Behar et al. [139], presented an approach to assess the ECG signal quality in regards to arrhythmia in the context of intensive care units. Similar approaches could also be utilized to assess the ECG quality during affect-related user studies.
3. **Photoplethysmogram Preprocessing:** A detailed description on PPG signal preprocessing methods applied to PPG data can be found in Elgendi [140] or Biswas et al. [141]. In order to remove motion artefacts, adaptive (filtering) approaches can be applied [142,143]. In more recent work, peak matching approaches in the spectral domain were employed to remove movement artefacts [144,145]. For the determination of RR intervals from identified R-peaks, similar algorithms as mentioned with ECG preprocessing can be applied. In addition, as shown by Li and Clifford [146], the quality of a PPG signal can be assessed using a combination of dynamical time warping and multilayer perceptron
4. **Electrodermal activity Preprocessing:** In order to remove artefacts from EDA data different approaches were presented. The approaches can be grouped into filtering and machine learning-based approaches. Only changes in the low-frequency domain of the EDA signal are physiologically plausible. Hence, low-pass filtering with a cut-off of, for example, 5 Hz [147] can be applied to remove high-frequency noise. After the noise removal, for example, Soleymani et al. [31], detrended the EDA signal by subtracting a moving average, computed on smoothed version of the signal. Machine learning-based approaches, using support vector machines or convex optimization, to identify and remove artefacts in EDA data can be found in Taylor et al. [148], Greco et al. [149]. As detailed in Section 3, the EDA signal consists of two components: A slowly varying baseline conductivity referred to as skin conductance level (SCL) and a series of peaks referred to as skin conductance response (SCR). In literature different approaches to separate these two components can be found: Benedek and Kaernbach [150], for instance, present an approach to separate SCL and SCR relying on nonnegative devolution. Alternatively, Choi et al. [56] utilized, a regularized least-squares detrending method, to separate the two components.
5. **Electromyogram Preprocessing:** Raw EMG data is often filtered to remove noise. For example, Wijsman et al. [69] report on a two step procedure. First, a bandpass filter, allowing frequencies from 20 to 450 Hz, was applied. Then, in order to remove residual power line interference from data, notch filters were applied. The notch filters attenuated the 50, 100, 150, 200, 250, and 350 Hz components of the signal. Cardiac artefacts are another common source of noise in EMG data. Hence, Willigenburg et al. [151] propose and compare different filtering procedures to remove ECG interference from the EMG signal.
6. **Respiration Preprocessing:** Depending on the signal quality, noise removal filtering techniques (e.g., bandpass filter with cut-off frequencies at 0.1 and 0.35 Hz) have to be applied. In addition, the raw respiration signal can be detrended by subtracting a moving average [86].

In the classical processing chain these preprocessing steps are followed by the **segmentation**. During this procedure the data is segmented using a sliding window of fixed size. The appropriate window size is crucial and depends on several aspects, such as the classification task or the considered sensor modality. Below appropriate choices for the window length of motion (ACC) and physiological data will be provided. In human activity recognition (HAR), ACC data is most frequently employed to detect activities and there exists a body of work, identifying appropriate window sizes for HAR [87,152,153]. A common finding is that in HAR the relevant patterns occur on short time scales. Therefore, window sizes of ∼5 s are common.

The time scales on which physiological responses to emotional stimuli occur are hard to define. Hence, considering physiological signals, finding an appropriate window size is difficult [87]. Moreover, due to inter-subject and inter-modality (e.g., ECG vs. EDA) differences, defining an appropriate window size becomes even more challenging. However, a meta analysis conducted by Kreibig [58] found that physiological features are commonly aggregated over fixed window lengths of 30 to 60 s.

### 5.2. Physiological Feature Extraction

Following the classical time series classification pipeline, features are computed on the segmented data. These features aggregate information present in the signal, and serve as inputs into the classifier. Extracted features can be grouped in various ways, such as time- or frequency-domain features, linear or non-linear features, unimodal or multimodal features, and so forth. Considering computational complexity, extracted features range from simple statistical features (e.g., mean, standard deviation) to often modality-dependent complex features (e.g., number of SCR peaks). Table 8 gives an overview of features commonly extracted and applied in the wearable-based AR literature. In the remainder of this section, we give a brief description of features commonly extracted from different wearable sensors. As mentioned previously electroencephalogram (EEG) and electrooculography are not in scope of this work and, hence, will not be detailed here. For a comprehensive review on EEG-based AR we refer the reader to Kim et al. [20].

#### 5.2.1. ACC-based Feature

From the HAR domain, a large set of ACC-based features is known. These features are often also employed in AR. Statistical features (mean, median, standard deviation, etc.) are often computed for each channel (x,y,z) separately and combined. Parkka et al. [154] showed that the absolute integral of acceleration can be used to estimate the metabolic equivalent of physical activities, which can be an interesting feature for affect recognition as well. Mozos et al. [45] used the first and second derivative of the accelerometer’s energy as feature, for example, to indicate the direction of change in activity level. Considering frequency-domain features, the power ratio of certain defined frequency bands, the peak frequency or the entropy of the power spectral density have been applied successfully.

#### 5.2.2. ECG- and PPG-based Features

From ECG and PPG data, various features related to cardiac activity are derived. Below, we provide a description of features commonly used in AR. For an in-depth analysis of features based on the cardiac cycle we refer to Malik [155]. Commonly the heart rate is used as feature. Based on the location of the R-peaks (or the systolic peak in the PPG signal) the inter beat interval (IBI) can be computed. The IBI serves as a new time series signal, from which various HRV features can be derived, both in *time- and frequency-domain*. For instance, from the IBI the number and percentage of successive RR intervals differing by more than a certain amount of time (e.g., 20 or 50 milliseconds) can be computed. These feature are referred to as NNX and pNNX, where X is the time difference threshold in milliseconds. Based on the Fourier-transformation of the IBI time series, various frequency-domain features can be computed, which reflect the sympathetic and parasympathetic activities of the autonomic nervous system. Four different frequency bands are established in this respect [96]. The ultra low frequency (ULF) and very low frequency (VLF) bands range from 0 to 0.003 Hz and from 0.003 to 0.03 Hz, respectively. Changes in low frequency (LF) band, ranged between 0.03 and 0.15 Hz, are mostly associated with the activity of the sympathetic nervous system (SNS). In contrast, the high frequency (HF) band, ranged from 0.15 to 0.4 Hz, is believed to reflect mostly the activity of the parasympathetic nervous system (PNS) [96]. Therefore, the LF/HF ratio quantizes is a descriptive feature indicating the influence of both, SNS and PNS, on the cardiac activity. In literature, for example, Healey and Picard [63], it was shown that the LF/HF ratio is a good indicator for stress. In addition to time and frequency domain-based features, *non-linear features* derived from ECG data were employed successfully wearable-based AR. Rubin et al. [96], for instance, presents a detailed description of non-linear ECG features (e.g., maximal Lyapunov exponent, standard deviations ($SD_{1}$ and $SD_{2}$) along major axes of a Poincaré plot, the $SD_{1}/SD_{2}$ ratio, sample entropy, etc.). Moreover, Valenza et al. [89], aiming to detect five levels of valence and arousal, compared the performance of linear and non-linear features. Their results indicate that non-linear features are able to improve classification scores significantly. Another class of features based on the cardiac cycle are referred to as *geometrical features*. An example is the triangular interpolation index [89,96,155]—A histogram of the RR intervals is computed, a triangular interpolation performed, and the baseline of the distribution is computed. Finally, the respiration is known to have an impact on the ECG signal. In literature, there exist different approaches for quantifying the effect of the RESP on the ECG data: Hovsepian et al. [95], for instance, employed the respiratory sinus arrhythmia (RSA), which is calculated by subtracting the shortest RR interval from the longest RR interval within one respiration cycle. In addition, Choi et al. [56] proposed a method of decomposing the HRV into a respiration- and a stress-driven component.

#### 5.2.3. EDA-Based Features

SCR SCL Considering the EDA signal, basic *statistical features* (e.g., mean, standard deviation, min, max) are commonly used [147]. In addition, Koelstra et al. [35] provides a list of statistical (e.g., average rising time and decay rate) and *frequency domain-based* (spectral power values in the 0–2.4 Hz frequency bands) EDA features. Furthermore, the EDA is known to consist of two components—the skin conductance level (SCL) and skin conductance response (SCR) component. Approaches to separate these components were, for instance, presented by Choi et al. [56] or Lim et al. [156]. The degree of linearity of the SCL component was shown to be a useful feature [56] . Considering the SCR component, the identified SCR segments are counted and further statistical features derived: sum of the SCR startle magnitudes and response durations, in the area under the identified SCRs [63]. The SCR-related features were found to be particularly interesting as they are closely linked to high arousal states [34].

#### 5.2.4. EMG-Based Features

From the EMG signal, various *time- and frequency-domain* features can be extracted. Christy et al. [159], working on the DEAP dataset, computed statistical features such as mean, median, standard deviation, and interquartile ranges on the EMG data. Other researchers used frequency-based features such as peak or mean frequencies [120,160]. Another frequently used feature is the signal energy of either the complete signal [35] or specific frequency ranges (e.g., 55–95 Hz, 105–145 Hz) [37]. Wijsman et al. [120] performed a reference voluntary contraction measurement to compute a personalised EMG gap feature. This feature is defined as the relative time the EMG amplitude is below a specific percentage of the amplitude of the reference measurements.

#### 5.2.5. Respiration-Based Features

Soleymani et al. [31] pointed out that slow respiration is linked to relaxation. In contrast, irregular and quickly varying breathing patterns correspond to more aroused states like, anger or fear [34,157]. Therefore, different respiration patterns can provide valuable information for the detection of affective states. Plarre et al. [46] describe a number of *time-domain* features which aggregate information about breathing cycles: breathing rate, inhalation (I) and exhalation (E) duration, ratio between I/E, stretch (the difference between the peak and the minimum amplitude of a respiration cycle), and the volume of air inhaled/exhaled. Considering *frequency-domain* features, Kukolja et al. [158] used mean power values of four frequency subbands (0–0.1 Hz, 0.1–0.2 Hz, 0.2–0.3 Hz, and 0.3–0.4 Hz) in order to classify different types of emotions. As discussed previously features relation cardiac and respiratory activities (like RSA are frequently employed [46,95].

#### 5.2.6. Temperature-Based Features

Changes in body temperature might be attributed to the ’fight or flight’ response (see Section 3). During this physiological state, the blood flow to the extremities is restricted in favour of an increased blood flow to vital organs. Hence, temperature-based features can be relevant indicators for a severe stress response. Gjoreski et al. [13], for instance, extract the mean temperature, the slope, and the intersection of a linear regression line with the y-axis as features.

### 5.3. Classification

In AR the classification is either done using statistical approaches (e.g., ANOVA) or machine learning (ML) methods (e.g., support vector machine (SVM), k-nearest neighbour (kNN)). For both types of analyses, features similar to the ones described in Section 5.2 are combined into a feature vector, associated with a label and used as inputs. Since statistical analysis plays only a minor role in wearable-based AR literature, we focus in this section on classification approaches utilising ML techniques. In Table 9 the same studies are presented as in Table 4. However, here we focus on the employed classification algorithms, number of target affective classes, setting of the study, number of participants, evaluation schemes, and achieved classification performance. The performance is, if possible, reported as *accuracy*, indicating the overall percentage of correctly classified instances. The rest of this section discusses and compares the different approaches and their performance.

The algorithm column in Table 9 indicates that the SVM is the most common classification algorithm. It is employed in 48% of the considered studies. This is to some degree surprising as the SVM requires careful adjustment of the kernel size γ and the trade-off parameter *C*. For this adjustment the recorded data has to be split into *training, validation*, and *test* sets. The best set of hyperparameters can be found by performing a grid-search [45,95], evaluating the current hyperparameter on the *validation set*. The performance of the final model is then evaluated on the *test set*. Hence, when using a SVM, it is important to report the final test error (and *not* the validation error). kNN and decision-tree (DT), are the second most popular classifiers both applied in 22% of the considered studies. kNN and DT require only little hyperparameter tuning and, hence, are applied (almost) in an off-the-shelf way. Concluding from Table 9, ensemble methods (e.g., random-forest or AdaBoost) are employed less frequently. This is astonishing as ensemble methods have been proven to be strong classifiers. Fernández-Delgado et al. [161] evaluated 179 classifiers on more than hundred different datasets and found that the random-forest family ‘is clearly the best family of classifiers’. In the wearable-based AR community, Rubin et al. [96] employed random-forests to detect *panic* and *pre-panic* states, reaching a 97% and 91% accuracy, respectively. In addition, boosting was found to be a strong classifier [161], and Leo Breiman even considered it to be the ‘best off-the-shelf classifier in the world’ [162]. Mozos et al. [45] applied the AdaBoost method to detect stress, reaching an accuracy of 94%. For a detailed description of random-forests we refer the reader to Breiman [163] and an introduction into boosting can be found in Freund et al. [164]. Fernández-Delgado et al. [161] also found neural networks (NN) to be among the top-20 classifiers. Haag et al. [77] and Jaques et al. [97] used NN, in the form of multi-layered perceptrons, to detect different affective states. Convolutional neural network (CNN) and long short-term memory-based classification techniques, which are becoming popular in the field of human activity recognition [165,166], have not found broad application in the domain of wearable-based AR domain yet. Martinez et al. [93] compare the performance of learned and hand-crafted features to detect the affective states *relaxation*, *anxiety*, *excitement*, and *fun*. The learned features were extracted using a set of convolutional layers, and the final classification step was performed using a single-layer perceptron. The experiments of Martinez et al. [93] indicate that learned features lead to an improved classification performance (compared to the hand crafted features).

Judging from Table 9, a binary classification tasks were pursued in most (52%) presented studies. This holds even for the cases where the study protocol aimed at eliciting different emotions. A frequent task, following for instance Agrafioti et al. [91] or Abadi et al. [37], is to distinguish between high/low valence/arousal using physiological data.

Considering the setting, three different types of studies are distinguished: *lab* (L), *field* (F), and *field with constraints* (FC) studies. Studies conducted in a vehicle on public roads are referred to as FC studies, as subjects are constrained in their movement. In addition, studies were subjects followed a specific (outdoor) path, for example, Kanjo et al. [75] are referred to as FC studies. Most, 31 out of 46, studies presented in Table 9, solely base their results on data recorded in a lab setting. The popularity of lab studies is easily explained: in lab studies the study protocol is designed to elicit a set of specific target affective states (see Section 4.1). Hence, the signal to noise ratio is much higher than in field studies. Furthermore, once the set of stimuli is chosen the same protocol is applied to multiple subjects, which makes lab studies very efficient. However, models trained on data gathered in constrained environments, are likely to exhibit a poor performance in an less constrained setting.

In order to overcome this, field studies have become more frequent over the past years. This ‘out of the lab and into the fray’ [87] is also related to recent advances in mobile sensor technology and the broad acceptance of smart devices (watches, phones, et.) among users. As wearable-based AR clearly aims to detect the affective state users in unconstrained environments, this trend is certainly desirable. Recent work aspiring to detect stress in lab and real life scenarios has for instance been conducted by Gjoreski et al. [13], Plarre et al. [46], Hovsepian et al. [95], Taylor et al. [100]. Their results indicate that stress detection, based on wearable-based data and context information, is feasible, even in mostly unconstrained settings.

Finally, considering the number of study participants there is a large variation: The results reported in Table 9 are based on data originating from a single subject up to 104 subjects. Clearly, a large and diversified subject pool is desirable. This would allow to develop generalized models for wearable-based AR.

Judging from Table 9, n-fold CV (n∈[3,5,10,20,40]) is frequently employed as validation method (28%). Following this method, the dataset is randomly partitioned into *n* equally sized subsets. Then, n−1 subsets are used for training and the remaining one for testing. This procedure is repeated *n* times. Hence, each of the *n* subsets is used exactly once as test set. In case the trained model requires hyperparameter tuning, part of the training data can serve as validation set in each iteration. If features are extracted on overlapping windows and n-fold CV is used as validation methods the results are often overoptimistic. This is due to the strong correlation between the features extracted from overlapping windows. Leave-One-Out (LOO) CV is also used in several studies listed in Table 9. This is a specific version of the n-fold CV procedure, where *n* is equals to the total number of available feature vectors. In the LOO case each feature vector is used once for testing. A slightly different type of validation was performed by Abadi et al. [37]: Leave-One-Trial-Out (LOTO) CV. During LOTO CV, the model is trained on the data of all subjects but leaving one trial/stimulus (e.g., video) aside. The trained algorithm is then evaluated on the left-out data, and the procedure is repeated for each trial. LOO, LOTO, and n-fold CV lead to subject-dependent results. In order to obtain an subject independent score, corresponding to a more realistic results for real-life deployment, leave-one-subject-out (LOSO) CV should be applied. For this purpose, the algorithm under consideration is trained on the data of all but one subject. The data of the left-out subject is then used to evaluate the trained model. Repeating this procedure for all subjects in the dataset gives a realistic estimate of the model’s generalisation properties on completely unseen data. As indicated by Table 9, nowadays LOSO CV is widely accepted and applied. From the results shown here, it can be concluded that using the LOSO validation method leads to lower classification scores than applying n-fold or LOO CV. However, only LOSO provides the information on how good the trained model is able to perform on completely unseen data (e.g., data of a new user). Hence, we recommend using this validation scheme.

SNS The affect and stress recognition approaches presented in Table 9 report accuracies between 40% and 95%. Due to the lack of benchmarking datasets, the results obtained in different studies are hard to compare. On average, the classification accuracies obtained using lab data are higher than the ones obtained in field study data. Hovsepian et al. [95], who conducted both a lab and a field study, report on a 92% mean accuracy in detecting stress based on lab data. However, when field data is considered, the accuracy drops to 62%. Moreover, Healey et al. [87] conducted a field study and trained different classifiers on the collected data but none of them was able to perform better than random guessing. This indicates that wearable-based AR in the field is very challenging. As indicated in Table 4, most studies were conducted recording multimodal datasets. This might be motivated by a recent review of D’mello and Kory [1], who pointed out that the classifiers relying on multimodal input reach on average higher classification scores than their unimodal counterparts. Considering the accuracy of classifiers detecting high/low arousal and high/low valence separately it becomes apparent, that arousal is classified more reliably [37,77,89,91]. High arousal states are, from a physiological point of view, directed by the sympathetic nervous system (SNS) (see Section 3). Physiological changes directed by the SNS are quite distinct (e.g., increased heart rate, sweat production, etc.). Hence, detecting high arousal states using these physiological indicators is a feasible task. In contrast, detecting changes in a subject’s valence based on physiological data is a more challenging.

The performance of standard ML classifiers depend strongly on the employed features. Hence, the benefits of a careful feature selection can be threefold:

1. Feature selection can help to improve classification results.
2. Feature selection identifies cost-effective and yet strong predictors.
3. It provides a better understanding of the processes generating the data [167].

According to Guyon and Elisseeff [167], feature selection methods are grouped into filter-based methods, wrappers, and embedded methods. Filter-based methods select a subset of features (e.g., based on statistical a criterion) and do not take the used classifier into account. Wrapper-based methods (e.g., sequential feature selection) treat the learning algorithm as black box and assess the quality of a subset of features based on the final classification score [167]. Finally, embedded methods perform variable selection during training. Hence, the selection is commonly specific to the used classifier [167]. Feature selection methods also find application in AR. Kim and André [34], for instance, perform feature selection to improve the classification. Valenza et al. [89] used Principal Component Analysis to project the features onto a lower dimensional space. This linear method has the advantage that the features are condensed with only a minimal loss of information. For a detailed review of feature selection methods see Guyon and Elisseeff [167].

## 6. Discussion And Outlook

Based on the previous sections, we would like to go one step further and identify key challenges and opportunities in wearable-based affect recognition (AR). We will focus on the following key challenges in this section: (a) valence detection; (b) hardware; (c) datasets; (d) algorithmic challenges; and (e) long-term reasoning.

**Valence detection**: From Section 2 and Section 3, the link between physiological changes and the arousal axis of the circumplex model became apparent. Hence, it is not surprising that approaches of stress detection and arousal assessment in Table 9 reach high accuracies. However, valence-related changes in human physiology are more subtle and, therefore, difficult to detect. This explains the lower accuracy of valence detection systems in Table 9. In some studies [35,37], facial expressions, which are directly connected to valence (e.g., smiles), were recorded using facial electromyogram. However, this procedure is not applicable in everyday life due to practical considerations. One possibility to improve the assessment of valence is to incorporate contextual data into the classification process. This contextual information can range from audio samples (e.g., detection laughter), information about the sleep qualit, to calendar meta data or text (e.g., emails/chat). Following for instance Sano et al. [122], the regularity of sleep and duration has a very strong impact on the mood of a person and is a strong feature to predict the morning mood.

**Hardware**: The setups used to record physiological data in affect recognition studies are often either watch-like (e.g., *Empatica E4* [168]), chest-belt (e.g., *AutoSense* [169]) or stationary devices (e.g., *BioPac systems* [170]). Recent progress in flexible electronics enabled the development of sensor patches (e.g., Vivalnk [171]) and epidermal electronics. In recent research, the potential of epidermal electronics measuring different electrophysiological signals, like electrocardiogram, electromyogram, and even electroencephalogram has been demonstrated [172,173]. Up to now patches and epidermal electronics have found little application in affect recognition (field) studies. Furthermore, sensors and processing units can be integrated into fabric (for a comprehensive summary see Reiss and Amft [174]). These technologies offer an increased wearing comfort, potential new measurement positions [175], and are, similar to smartwatches, only minimally intrusive. Hence, they certainly deserve more attention in wearable-based affect recognition. In addition to the traditionally employed set of modalities (electrocardiogram, electrodermal activity, etc.), the merits of other sensors should be explored. First, considering the cardiac system, stress has been related to changes in blood pressure [176]. Hence, incorporating data representing a blood pressure correlate (e.g., pulse wave transit time [177]) could enable more reliable stress detectors. Second, body microphones placed on the subject’s chest or abdomen could provide further insights into the cardiac [178], respiration, and digestive system. Third, the chemical composition of perspiration could provide further information about the physiological state of a person. Hence, integrating chemical-electrophysiological sensors [179,180] in affect recognition studies has the potential to create new insights into the physiology of affective states. Finally, as already mentioned above, contextual information about the user could help to improve the classification. The sources of contextual data are nearly unlimited and range from ambient audio data to video streams provided by devices like smartglasses (e.g., Google Glass). These sources could be used to classify the surroundings of a user and the affective state of other nearby persons as well.

**Datasets**: The wearable-based affect recognition community lacks publicly available datasets, frequently used for benchmarking. In order to generate statistically meaningful results, a representative cohort of subjects is desirable. However, most affect recognition studies target students or research staff, which are likely to represent a homogeneous group [181]. In order to mitigate this selection bias, studies could recruit subjects from different social groups (gender, age, etc.). In addition, cross-cultural data collection would be very interesting in order to facilitate universal wearable-based affect recognition. Furthermore, as shown by Grünerbl et al. [182] or Rubin et al. [96], affect recognition systems can find application in clinical settings. However, in order to facilitate this type of application, datasets containing data from patients with specific health conditions are required. The available datasets (see Section 4.3) already feature multiple modalities. Measuring physiological changes in a redundant fashion (e.g., using electrocardiogram and photoplethysmogram) or using the same modality on various locations (e.g., wrist and torso) would facilitate a direct comparison of the signals. Studies on wearable/based emotion detection commonly elicit and detect multiple emotional states [12,35]. In contrast, stress detection systems mainly target binary problems (*stress* versus *no-stress*). In our opinion, robust affect recognition systems should be trained on datasets like WESAD [64], which include redundant data streams and different affective state (*stress, amusement*, and *neutral*). Up-to-date affect recognition research based on wearables mainly focus on lab studies. For benchmarking and exploitative studies, lab data is a good starting point. Hence, we hope that the observed trend towards field studies (see Table 9) continues. To support this trend, we provided in Section 4.2.1 practical guidelines on ground truth generation in field studies.

**Algorithmic challenges**: The way humans perceive and react to an affective stimulus is subject dependent. This highlights the importance of personalisation. However, the current state-of-the-art in wearable-based affect recognition makes little use of personalisation methods. One way to account for the subjective nature of affective states is to utilise online learning. Following this idea, a general model could be deployed, which is then customised. Customisation could happen, for instance, via an active labelling approach, where the user is occasionally asked to provide labels. In addition, semi-supervised or even unsupervised training methods could be used. To the best of our knowledge, these methods have not found application in wearable-based affect recognition research yet. Healthy subjects are unlikely to exhibit strong swings over the entire affective spectrum. Hence, in order to identify the rare extreme cases methods from anomaly detection could be applied [183]. In most studies presented in Table 9 classical feature-based machine learning algorithms (e.g., support vector machine, k-nearest neighbour, etc.) were employed. In human activity recognition [184], audio analysis [185] or stock return forecasting [186], which all deal with time series data, (deep) neural networks (NN) proved to be powerful classifiers. Using convolutional neural network (CNN) makes feature engineering obsolete, as via backpropagation features are learned. From a methodical point of view CNN offer interesting approaches to transfer [187] or semi-supervised [188] learning. Deep NN require a large amount of training data and are known to be resource intensive. Hence, deployment on an embedded device is an open research question. However, first approaches to deploy such models on embedded devices were presented in Reference [189]. Hence, due to strong interest in NN from both academia and industry, we are confident that resource-related issues will be solved in near future.

**Long-term reasoning**: Image-based affect recognition systems can only perform a temporal- and spatial-limited assessment of the user’s state (e.g., while driving [16]). In contrast, wearable-based affect recognition systems detect the user’s affective state continuously and ubiquitously. This can be used for a deeper analysis, providing reasoning for certain affective states or behavioural patterns. First approaches of long-term reasoning were presented by Gjoreski et al. [13] and in the HappyMeter App [190]. The latter investigated correlations between affective states and environmental conditions (e.g., temperature, wind, humidity) or persons nearby. Visualizing this information can increase awareness of specific situations (e.g., showing locations where the user is stressed). Essential for this correlation analysis, is contextual information. We see a large potential for this research direction, as the reasoning methods presented above are still in an early stage.

The aim of this review was to provide a broad overview and in-depth understanding of the theoretical background, methods, and best practices of wearable-based affect recognition. Currently, there is a strong trend to small, lightweight, affordable, and wearable electronic gadgets. These devices can be used for sensing, storing, and data processing [18]. Hence, they offer an ideal platform for enhanced affect recognition systems. There is a wide range of applications for such systems, in particular in the consumer and healthcare domain. From a healthcare point of view, wearable-based affect recognition systems could, for instance, help to ubiquitously monitor the state of patients with mental disorders (e.g., depression). This data could provide valuable insights for therapists, promoting behaviour change interventions [191]. Furthermore, these systems could facilitate the development of tele-mental [192] and tele-medical applications. Wearable-based affect recognition systems could improve self monitoring, provide users with a better understanding of their affective states, and support behavioural changes. Beyond these health-related applications, affect recognition systems could be used in urban planning [193] or to improve human-machine interfaces. Despite the impressive progress made in recent years, the applications mentioned above are still under research and not available for customers. We are convinced that robust and personalised affect recognition systems applicable in everyday life could provide many users with an added value. Hence, we encourage the community to support and address the remaining challenges.

## Figures and Tables

**Table 1:** Major functions of the sympathetic nervous system and parasympathetic nervous system.

Sympathetic Nervous System (SNS)	Parasympathetic Nervous System (PNS)
associated with ‘fight or flight’	associated with ‘rest and digest’
pupils dilate	pupils constrict
decreased salivation and digestion	increased salivation and digestion
increased heart and respiration rate	decreased heart and respiration rate
increased electrodermal activity	
increased muscle activity	
adrenalin and glucose release	

**Table 2:** Four exemplary affective states and their physiological response [58]. Abbreviations: ↓ indicate a decrease, ↑ indicates an increase, ↑↓ indicate both increase and decrease (depending on the study), − indicates no change in the parameter under consideration, # represents number of.

	Anger	Sadness	Amusement	Happiness
		(Non-Crying)		
**Cardiovascular:**				
HR	↑	↓	↑↓	↑
HRV	↓	↓	↑	↓
**Electrodermal:**				
SCL	↑	↓	↑	↑−
# SCRs	↑	↓	↑	↑
**Respiration:**				
Respiration rate	↑	↑	↑	↑

**Table 3:** Sensor modalities and derived indicators used in the wearable-based AR. Abbreviations: heart rate (HR), heart rate variability (HRV).

	Physiological Signal Type	Derived Indicators
**Head/Face**	Electroencephalogram	Electric potential changes of brain neurons
	Electromyogram	Facial muscle activity (e.g., zygomaticus major)
	Electrooculography	Eye movements
	Photoplethysmogram (ear)	HR and HRV
**Torso/Back**	Electrocardiogram	HR and HRV
	Electrodermal activity	Tonic and phasic component
	Electromyogram	Muscle activity
	Inertial sensor	Physical activity/body pose
	Respiratory inductive Plethys-mograph	Respiration rate and volume
	Body thermometer	Temperature
**Hand/Wrist**	Electrodermal activity meter	Tonic and phasic component
	Blood Oxymeter	Blood oxygen saturation
	Blood pressure	Sphygmomanometer
	Inertial sensor	Physical activity
	Photoplethysmogram	HR and HRV
	Thermometer	Temperature
**Feet/Ankle**	Electrodermal activity	Tonic and phasic component
	Inertial sensor	Physical activity
Context	Sensors of a mobile phone (GPS, microphone, etc.)	Location, Sound, Activity, Interaction

**Table 4:** Affective states and sensor signals frequently employed in wearable-based AR. Table 9 provides further detail on algorithms, location and performance. Abbreviations: 3-axes acceleration (ACC), blood pressure (BP), (EEG), electromyogram (EMG), electrooculography (EOG), heart rate (HR), magnetoencephalogram (MEG), pupil diameter (PD), photoplethysmogram (PPG), respiration (RESP), skin-temperature (TEMP), arterial oxygen level (SpO2), low arousal/low valence (LALV), low arousal/high valence (LAHV), high arousal/low valence (HALV), high arousal/high valence (HAHV)

	Author	Affective States	Sensor Signals
<2005	Picard et al. [12]	Neutral, anger, hate, grief, joy, platonic/romantic love, reverence	EDA, EMG, PPG, RESP
	Haag et al. [77]	Low/medium/high arousal and positive/negative valence	ECG, EDA, EMG, TEMP, PPG, RESP
	Lisetti and Nasoz [70]	Sadness, anger, fear, surprise, frustration, amusement	ECG, EDA, TEMP
2005	Liu et al. [78]	Anxiety, boredom, engagement, frustration, anger	ECG, EDA, EMG
	Wagner et al. [79]	Joy, anger, pleasure, sadness	ECG, EDA, EMG, RESP
	Healey and Picard [63]	Three stress levels	ECG, EDA, EMG, RESP
07	Leon et al. [80]	Neutral/positive/negative valence	EDA, HR, BP
2008	Zhai and Barreto [81]	Relaxed and stressed	EDA, PD, PPG, TEMP
	Kim et al. [82]	Distinguish high/low stress group of individuals	PPG
	Kim and André [34]	Four quadrants in valence-arousal space	ECG, EDA, EMG, RESP
	Katsis et al. [83]	High/low stress, disappointment, euphoria	ECG, EDA, EMG, RESP
2009	Calvo et al. [84]	Neutral, anger, hate, grief, joy, platonic/romantic love, reverence	ECG, EMG
	Chanel et al. [85]	Positively/negatively excited, calm-neutral (in valence-arousal space)	BP, EEG, EDA, PPG, RESP
	Khalili and Moradi [86]	Positively/negatively excited, calm (valence-arousal space)	BP, EEG, EDA, RESP, TEMP
2010	Healey et al. [87]	Points in valence arousal space. moods	ACC, EDA, HR, audio
2011	Plarre et al. [46]	Baseline, different types of stress (social, cognitive and physical), perceived stress	ACC, ECG, EDA, RESP, TEMP, ambient temperature
	Hernandez et al. [88]	Detect stressful calls	EDA
2012	Valenza et al. [89]	Five classes of arousal and five valence levels	ECG, EDA, RESP
	Hamdi et al. [90]	Joy, sadness, disgust, anger, fear, surprise	ECG, EEG, EMG
	Agrafioti et al. [91]	Neutral, gore, fear, disgust, excitement, erotica, game elicited mental arousal	ECG
	Koelstra et al. [35]	Four quadrants in valence-arousal space	ECG, EDA, EEG, EMG, EOG, RESP, TEMP, facial video
	Soleymani et al. [31]	Neutral, anxiety, amusement, sadness, joy, disgust, anger, surprise, fear	ECG, EDA, EEG, RESP, TEMP
2013	Sano and Picard [92]	Stress vs. neutral	ACC, EDA, phone usage
	Martinez et al. [93]	Relaxation, anxiety, excitement, fun	EDA, PPG
2014	Valenza et al. [36]	Four quadrants in valence-arousal space	ECG
	Adams et al. [94]	Stress vs. neutral (aroused vs. non-aroused)	EDA, audio
2015	Hovsepian et al. [95]	Stress vs. neutral	ECG, RESP
	Abadi et al. [37]	High/Low valence, arousal and dominance	ECG, EOG, EMG, near-infrared face video, MEG
2016	Rubin et al. [96]	Panic attack	ACC, ECG, RESP
	Jaques et al. [97]	Stress, happiness, health values	EDA, TEMP, ACC, phone usage
	Rathod et al. [98]	Normal, happy, sad, fear, anger	EDA, PPG
	Zenonos et al. [29]	Excited, happy, calm, tired, bored, sad, stressed, angry	ACC, ECG, PPG, TEMP
	Zhu et al. [99]	Angle in valence arousal space	ACC, phone context
	Birjandtalab et al. [76]	Relaxation, different types of stress (physical, emotional, cognitive)	ACC, EDA, TEMP, HR, SpO2
2017	Gjoreski et al. [13]	Lab: no/low/high stress; Field: stress vs. neutral	ACC, EDA, PPG, TEMP
	Mozos et al. [45]	Stress vs. neutral	ACC, EDA, PPG, audio
	Taylor et al. [100]	Tomorrow’s mood, stress, health	ACC, EDA, context
	Girardi et al. [101]	High vs. low valence and arousal	EEG, EDA, EMG
2018	Schmidt et al. [64]	Neutral, amusement, stress	Torso: ACC, ECG, EDA, EMG, RESP, TEMP; Wrist: ACC, EDA, PPG, TEMP
	Zhao et al. [102]	LALV, LAHV, HALV, HAHV	EDA, PPG, TEMP
	Marín-Morales et al. [103]	LALV, LAHV, HALV, HAHV	ECG, EEG
	Santamaria- Granados et al. [104]	LALV, LAHV, HALV, HAHV	ECG, EDA
2019	Heinisch et al. [67]	High positive pleasure high arousal, high negative pleasure high arousal and neutral	EMG, PPG, TEMP
	Hassan et al. [105]	Happy, relaxed, disgust, sad and neutral	EDA, PPG, EMG (from DEAP)
	Kanjo et al. [75]	Five valence classes	ACC, EDA, HR, TEMP, environmental, GPS
	Di Lascio et al. [66]	Detect laughter episodes	ACC, EDA, PPG

**Table 5:** Questionnaires utilized in recent wearable-based AR field studies. Abbreviations: Number of Items (I), Big Five Inventory (BFI), Photo Affect Meter (PAM), Positive and Negative Affect Schedule (PANAS), PHQ-9, Pittsburgh Sleep Quality Index (PSQI), Perceived Stress Scale (PSS), Self-Assessment Manikins (SAM), Stress Response Inventory (SRI), Stait-Trait Anxiety Inventory (STAI).

Questionnaires Employed *Prior or After* the Study.				
**Goal**	**Tool and Description**	**I**	**Source**	**Example Use**
Stress level	PSS: subject’s perception and awareness of stress	10	Cohen et al. [123]	Sano and Picard [92]
	SRI: score severity of stress-related symptoms within time interval	22	Koh et al. [124]	Kim et al. [82]
Depression level	PHQ-9: score DSM-IV manual	9	Kroenke et al. [125]	Wang et al. [9]
Loneliness level	UCLA loneliness scale: addressing loneliness and social isolation.	20	Russell [126]	Wang et al. [9]
Sleep behaviour and quality	PSQI: Providing information about sleep quality	19	Buysse et al. [127]	Sano and Picard [92]
Measure suc-cess areas	Flourishing scale: measure success, self-esteem, purpose and optimism	8	Diener et al. [128]	Wang et al. [9]
Personality traits	BFI: indicating personality traits	44	John and Srivastava [129]	Taylor et al. [100], Sano et al. [122]
**Questionnaires employed in ecological-momentary-assessment (during study).**				
Affect in Valence-arousal space	Mood Map: a translation of the circumplex model of emotion	2	Morris and Guilak [130]	Healey et al. [87]
	SAM	2	Morris [38]	Schmidt et al. [64]
Positive and negative affect	Shortened PANAS	10	Muaremi et al. [74]	Muaremi et al. [74]
Positive Affect of PANAS	PAM: choose one of 16 images, mapped to the valence-arousal space	1	Pollak et al. [131]	Wang et al. [9]
Subjective mood indicator	Smartphone app querying user’s mood	8	HealthyOffice app	Zenonos et al. [29]
Stress level assessment	Adaptation of PSS for ambulatory setting	5	Hovsepian et al. [95]	Hovsepian et al. [95]
	Log current Stress Level	1	Gjoreski et al. [13] Hernandez et al. [88]	Gjoreski et al. [13] Hernandez et al. [88]
Severity of panic attack symptoms	Symptoms from the DSM-IV and Panic Disorder Severity Scale standard instrument	15	Shear et al. [132]	Rubin et al. [121]

**Table 6:** Questionnaires employed during recent field studies, focusing on the applied scheduling (Pre-, During or Post-study).

	Author	Employed Questionnaires and Their Scheduling
Emotion	Healey et al. [87]	*During study:* Participants completed EMAs whenever they felt a change in their affective/physiological state. EMAs included a form of the circumplex model and a field for free text. Conducted Interviews at the end of each workday to generate additional labels and revision.
	Rubin et al. [121]	*During study:* Start/stop time and severity ratings of 15 panic attack symptoms were reported by the subject using a mobile app.
	Jaques et al. [97]	*During study:* Students reported health, stress and happiness twice a day (morning and evening).
Stress	Hernandez et al. [88]	*During study:* Nine employees of a call center rated all their incoming calls on a 7 point likert scale (endpoints marked as “extremely good/bad”).
	Muaremi et al. [74]	*During:* Participants were asked to fill in a shortened PANAS four times between 8 a.m and 8 p.m. Before going to sleep they answered the question: “How stressful have you felt today?”
	Kim et al. [82]	*Pre-study:* In order to divide the subjects into two groups they filled out a simplified SRI.
	Sano and Picard [92]	*Pre-study:* Participants filled in a PSS, PSQI, and BFI. *During study:* Morning/evening EMAs on sleep, mood, stress level, health, and so forth. *Post-study:* Participants filled in questionnaires on health, mood, and stress.
	Adams et al. [94]	*Pre-study:* Participants completed a PANAS, PSS, and a measure of mindfulness. *During study:* Self-reports approximately every 30 min. (with small random variations). Participants reported on momentary stress and affect. Additional reports and a small free text field were available too. *Post-study:* Semi-structured interview at the end of the end data collection.
	Hovsepian et al. [95]	*During study:* EMAs randomly scheduled approximately 15 times. During each EMA subjects filled in a shortened version of the PSS containing 6 items.
	Gjoreski et al. [13]	*During study:* Subjects replied to 4 to 6 randomly scheduled EMAs. During each EMA subjects reported on their current stress level.
	Schmidt et al. [64]	*Pre-Study:* PSS and PSQI *During study:* EMAs were scheduled every 2 h (with small random variations) during the wake time of the subjects. EMAs included valence+arousal SAM, basic emotions, stress level, shortened STAI, and PAM.
Mood	Wang et al. [9]	*Pre-study:* Subject filled in a number of behavioural and health surveys. *During study:* Every participant filled in 8 EMAs every day. The EMAs include measures on mood, health, stress and other affective states. *Post-study:* Interviews and the same set of behavioural and health surveys were administered.
	Sano et al. [122]	*Pre-study:* subjects filed BFI, PSQI, and Morningness-Eveningness [133] questionnaire. *During study:* similar to Sano and Picard [92] subject filled EMAs in morning and evening reporting on: activities, sleep, social interaction, health,mood, stress level and tiredness. *Post-study:* Subjects filed in a PSS, STAI, and other questionnaires related to physical and mental health.
	Zenonos et al. [29]	*During study:* EMAs were scheduled every two hours. For the EMAs an app was used, containing sliders from 0-100 for 8 moods. Additionally, a free text field was provided.

**Table 7:** Publicly available datasets relevant for wearable affect and stress recognition. Abbreviations: Number of subjects (Sub), Location (Loc), Lab (L), Field (F), Field with constraint (FC), Population (Pop) reported as mean age or as category, College Student (CS), Graduate Student (GS), 3-axes acceleration (ACC), electrocardiogram (ECG), electrodermal activity (EDA), electroencephalogram (EEG), electromyogram (EMG), electrooculography (EOG), magnetoencephalogram (MEG), respiration (RESP), arterial oxygen level (SpO2), skin-temperature (TEMP).

	Name	Labels	Pop.	Sub.	Loc.	Included Modalities
Emotion (E)	Eight-Emotion [12]	Neutral, anger, hate, grief, joy, platonic love,romantic love, reverence	GS	1	L	ECG, EDA, EMG, RESP
	DEAP [35]	Continuous scale of valence, arousal, liking, dominance, Discrete scale of familiarity	26.9	32	L	ECG, EDA, EEG, EMG, EOG, RESP, TEMP, face video (not all subjects)
	MAHNOB-HCI [31]	Discrete scale of valence, arousal, dominance, predictability, Emotional keywords	26.06	27	L	ECG, EDA EEG, RESP, TEMP, face and body video, eye gaze tracker, audio
	DECAF [37]	Discrete scale of valence, arousal, dominance	27.3	30	L	ECG, EMG, EOG, MEG, near-infrared face video
	ASCERTAIN [39]	Discrete scale of valence, arousal, liking, engagement, familiarity, Big Five	30	58	L	ECG, EDA, EEG, facial activity data (facial landmark trajectories)
	USI_Laughs [66]	Detect and distinguish laughter from other events	26.70	34	L	ACC, EDA, PPG, TEMP
Stress (S)	Driver [63]	Stress levels: low, medium, high	-	24	FC	ECG, EDA, EMG, RESP
	Non-EEG [76]	Four types of stress (physical, emotional, cognitive, none)	CS	20	L	ACC, EDA, HR, TEMP, SpO2
	Distracted Driving [134]	Driving being subject to no, emotional, cognitive, and sensorimotor distraction	Elder + Young	68	L	EDA, heart and respiration rate, facial expressions, eye tracking
	StudentLife [9]	Sleep, activity, sociability, mental well-being, stress, academic performance	CS + GS	48	F	ACC, audio, context, GPS, smartphone usage
E+S	WESAD [64]	Three affective states: neutral, amusement, stress	27.5	15	L	**chest**: ACC, ECG, EDA, EMG, RESP, TEMP; **wrist**: ACC, EDA, PPG, TEMP

**Table 8:** Features commonly extracted and applied in the wearable-based AR.

	Features
**ACC**	**Time-domain:** Statistical features (e.g., mean, median, standard deviation, absolute integral, correlation between axes), first and second derivative of acceleration energy **Frequency-domain:** Power ratio (0–2.75 Hz and 0–5 Hz band), peak frequency, entropy of the normalised power spectral density **References:** [45,137,153,154]
**ECG/PPG**	**Time-domain:** Statistical features (e.g., mean, median, 20th and 80th percentile), HR, HRV, statistical features on HRV (e.g., Root Mean Square of Successive Differences (RMSSD), Standard Deviation of the RR Intervals (SDNN)), number and percentage of successive RR intervals differing by more than 20 ms (NN20, pNN20) or 50 ms (NN50, pNN50), pNN50/pNN20 ratio, **Frequency-domain:** Ultra low (ULF, 0–0.003 Hz), very low (VLF, 0.003–0.03 Hz), low (LF, 0.03–0.15 Hz), and high (HF, 0.15–0.4 Hz) frequency bands of HRV, normalised LF and HF, LF/HF ratio **Non-linear:** Lyapunov exponent, standard deviations ($SD_{1}$ and $SD_{2}$) from Poincaré plot, $SD_{1}/SD_{2}$ ratio, sample entropy **Geometrical:** triangular interpolation index **Multimodal:** respiratory sinus arrhythmia, motion compensated HR , respiration-based HRV decomposition **References:** [56,63,89,95,96,155]
**EDA**	**Time-domain:** Statistical features (mean, standard deviation, min, max, slope, average rising time, mean of derivative, etc.) **Frequency-domain**: 10 spectral power in the 0–2.4 Hz bands **SCL features**: Statistical features, degree of linearity **SCR features**: Number of identified SCR segments, sum of SCR startle magnitude and response durations, area under the identified SCRs **References:** [56,63,147,148,149,156]
**EMG**	**Time-domain:** Statistical features, number of myoresponses **Frequency-domain:** Mean and median frequency, energy **References:** [34,35,69]
**RESP**	**Time-domain:** Statistical features (e.g., mean, median, 80th percentile) applied to: inhalation (I) and exhalation (E) duration, ratio between I/E, stretch, volume of air inhaled/exhaled **Frequency-domain:** Breathing rate, mean power values of four subbands (0–0.1 Hz, 0.1–0.2 Hz, 0.2–0.3 Hz and 0.3–0.4 Hz) **Multimodal:** RSA **References:** [34,46,95,157,158]
**TEMP**	**Time-domain:** Statistical features (e.g., mean, slope), intersection of the y-axis with a linear regression applied to the signal **References:** [13,113]

**Table 9:** Comparison of algorithms, validation methods, and accuracies of recent wearable-based AR studies. If not stated differently, scores are reported as (mean) accuracy. Abbreviations: Setting (Set.), Lab (L), Field (F), Field with constraint (FC), Validation (Val), cross-validation (CV), Leave-One-Out (LOO), leave-one-subject-out (LOSO), Leave-One-Trial-Out (LOTO), Arousal (AR), Valence (VA), Dominance (DO), Liking (LI), AdaBoost (AB), Analysis of Variance (ANOVA), Bayesian Network (BN), CNN, deep belief network (DBN), Gradient Boosting (GB), Gaussian Mixture Model (GMM), Hidden Markow Model (HMM), linear discriminant analysis (LDA), Linear Discriminant Function (LDF), Logistic Regression (LR), Naive Bayes (NB), NN, Passive Aggressive Classifier (PA), RF, Decision/Regression/Function Tree (DT/RT/FT), Ridge Regression (RR), Quadratic Discriminant Analysis (QDA).

	Author	Algorithm	Classes	Set.	Sub.	Val.	Accuracy
<2005	Picard et al. [12]	kNN	8	L	1	LOO	81%
	Haag et al. [77]	NN	contin.	L	1	3-fold split	AR: <96%, VA: <90%
	Lisetti and Nasoz [70]	kNN, LDA, NN	6	L	14	LOO	72%; 75%; 84%
2005	Liu et al. [78]	BN, kNN, RT, SVM	5	L	15	LOO	74%; 75%; 84%; 85%
	Wagner et al. [79]	kNN, LDF, NN	4	L	1	LOO	81%; 80%; 81%
	Healey and Picard [63]	LDF	3	FC	24	LOO	97%
07	Leon et al. [80]	NN	3	L	8+1	LOSO	71%
2008	Zhai and Barreto [81]	DT, NB, SVM	Bin.	L	32	20-fold CV	88%; 79%; 90%
	Kim et al. [82]	LR	Bin.	FC	53	5-fold CV	∼63%
	Kim and André [34]	LDA	4	L	3	LOO	sub. dependent/independent: 95%/70%
	Katsis et al. [83]	SVM	4	L	10	10-fold CV	79%
2009	Calvo et al. [84]	BN, FT, LR, NB, NN, SVM	8	L	3	10-fold CV	one subject: 37–98%, all subjects: 23–71%
	Chanel et al. [85]	LDA, QDA, SVM	3/Bin.	L	10	LOSO	<50%; <47%; <50%, Bin. <70%
	Khalili and Moradi [86]	QDA	3	L	5	LOO	66.66%
10	Healey et al. [87]	AB,DT, BN, NB	Bin.	F	19	10-fold CV	None ^2^
2011	Plarre et al. [46]	AB, DT, SVM/ HMM	Bin.	L/F	21/17	10-fold CV	82%; 88%; 88%/0.71 ^3^
	Hernandez et al. [88]	SVM	Bin.	F	9	LOSO	73%
2012	Valenza et al. [89]	QDA	5	L	35	40-fold CV	>90%
	Hamdi et al. [90]	ANOVA	6	L	16	-	None ^4^
	Agrafioti et al. [91]	LDA	Bin.	L	31	LOO	Active/Pas AR: 78/52% Positive/Neg VA: <62%
	Koelstra et al. [35]	NB	Bin.	L	32	LOO	AR/VA/LI: 57%/63%/59%
	Soleymani et al. [31]	SVM	3	L	27	LOSO	VA: 46%, AR: 46%
2013	Sano and Picard [92]	kNN, SVM	Bin.	F	18	10-fold CV	<88%
	Martinez et al. [93]	CNN	4 ^1^	L	36	3-fold CV	learned features: <75%, hand-crafted: <69%
2014	Valenza et al. [36]	SVM	Bin.	L	30	LOO	VA: 79%, AR: 84%
	Adams et al. [94]	GMM	Bin.	F	7	-	74%
2015	Hovsepian et al. [95]	SVM/BN	Bin.	L/F	26/20	LOSO	92%/>40%
	Abadi et al. [37]	NB, SVM	Bin.	L	30	LOTO	VA/AR/DO: 50-60%
2016	Rubin et al. [96]	DT, GB, kNN, LR, PA, RF, RR, SVM	Bin.	F	10	10-fold CV	Bin. panic: 73–97% Bin. pre-panic: 71–91%
	Jaques et al. [97]	LR, NN,SVM	Bin.	F	30	5-fold CV	<76%; <86%; <88%
	Rathod et al. [98]	Rule-based	6	L	6	-	<87%
	Zenonos et al. [29]	DT, kNN, RF	5	F	4	LOSO	58%; 57%; 62%
	Zhu et al. [99]	RR	1	F	18	LOSO	$0.24\pi \approx 43^{\circ }$ ^5^
	Birjandtalab et al. [76]	GMM	4	L	20	-	<85%
2017	Gjoreski et al. [13]	AB, BN, DT, kNN, RF, SVM	3/Bin.	L/F	21/5	LOSO	<73%/<90%
	Mozos et al. [45]	AB, kNN, SVM	Bin.	L	18	CV	94%; 93%; 87%
	Taylor et al. [100]	Single/Multitask LR, NN, SVM	Bin.	F	104	Cust. ^6^	Mood: <78%, Stress/Health<82%
	Girardi et al. [101]	DT, NB, SVM	Bin.	L	19	LOSO	$F1_{AR/VA}<63.8/58.5\%$
2018	Schmidt et al. [64]	AB, DT, kNN, LDA, RF	3/Bin.	L	15	LOSO	<80%/<93%
	Zhao et al. [102]	NB, NN, RF, SVM	4/Bin.	L	15	LOSO	76%
	Marín-Morales et al. [103]	SVM	Bin.	L	60	LOSO	Val<75%, AR<82%
	Santamaria- Granados et al. [104]	CNN	Bin.	L	40	-	Val: 75%, AR:71%
2019	Heinisch et al. [67]	DT, kNN, RF	3	L	18	LOSO	<67%
	Hassan et al. [105]	DBN+SVM	5	L	32	10-fold CV	89.53% use DEAP
	Kanjo et al. [75]	CNN+LSTM	5	FC	34	User ^7^	<95%
	Di Lascio et al. [66]	LR, RF, SVM	Bin.	L	34	LOSO	<81%

^1^ Given as pairwise preferences. ^2^ DT overfit, other classifiers performed worse than random guessing. ^3^ Correlation between self-reported and output of model. ^4^ No significant differences could be found between the affective states. ^5^ Mean absolute error of mood angle in circumplex model. ^6^ 80/20% split of the entire data+5-fold CV. ^7^ User specific models. Trained random on 70/30% splits with non-overlapping windows.
